# Characterization of the metabolome and proteome of stem cell-derived human primordial germ cell-like cells: a multi-omics approach

**DOI:** 10.1242/bio.062801

**Published:** 2026-09-18

**Authors:** Madalena Vaz Santos, Bauke V. Schomakers, Marta Llobet Ayala, Tara Jamali, Michel van Weeghel, Ans M. M. van Pelt, Callista L. Mulder, Geert Hamer

**Affiliations:** ^1^ Reproductive Biology Laboratory, Center for Reproductive Medicine, Amsterdam UMC, location University of Amsterdam, 1105 AZ Amsterdam, The Netherlands; ^2^ Amsterdam Reproduction and Development Research Institute, 1105 AZ Amsterdam, The Netherlands; ^3^ Laboratory Genetic Metabolic Diseases, Amsterdam UMC, location University of Amsterdam, 1105 AZ Amsterdam, The Netherlands; ^4^ Core Facility Metabolomics, Amsterdam UMC, University of Amsterdam, 1105 AZ Amsterdam, The Netherlands

**Keywords:** Human, Primordial germ cells, Induced pluripotent stem cells, Metabolome, Proteom, *In vitro*

## Abstract

Primordial germ cells (PGCs) are the population of cells that, in the human embryo, are initially specified at day 12 post-fertilization and form the precursor cells for future gametes. Although *in vitro* differentiation of PGCs from human stem cells has been achieved, these human PGC-like cells (hPGCLCs) fail to completely mature without the use of *ex vivo* human or animal gonadal soma. Previous studies in mice revealed that several metabolic changes occur during the specification and maturation of these cells, which are essential for their developmental progress. However, little is known about the metabolic profile of human PGCs. Due to the scarcity of human PGCs, particularly at the early specification stage, hPGCLCs serve as a research model to study PGC formation. To characterize the metabolic and proteomic profile of these cells, we differentiated hPGCLCs using induced pluripotent stem cells and performed a mass spectrometry analysis to establish their metabolome and proteome. These cells revealed a distinct metabolic profile, with changes particularly evident at the proteome level. This included a shift between canonical and noncanonical citric acid cycles in hPGCLCs, downregulation of late-stage glycolysis and reduction of nucleotide *de novo* synthesis. By providing an integrative map of these metabolic networks, we aim to provide insight into the metabolism of hPGCLC development that could help improve methods for fully *in vitro* differentiation and maturation of hPGCLCs.

## Teaser

**Summary:** Metabolic network analysis of human primordial germ cell-like cell development *in vitro* reveals a distinct profile compared with iPSCs and non-hPGCLCs, most evident at the proteome level.

## INTRODUCTION

Major breakthroughs have been achieved in recent years in the emerging field of *in vitro* gametogenesis (IVG), particularly in the generation of gametes from stem cells in the laboratory. In mice, both functional oocytes and round spermatids have been generated from embryonic and induced pluripotent stem cells (iPSCs) (Morohaku et al., 2016; Hayashi et al., 2017b; Sun et al., 2018; Ishikura et al., 2021; Lari et al., 2026). These advances have sparked major practical and ethical discussions regarding possible clinical applications in the fields of fertility preservation and treatment (de Bruin et al., 2025).

However, with only embryonic stem cells or iPSCs, *in vitro* human gametogenesis has not been achieved. Primordial germ cells (PGCs) are the population of cells that arise in the human embryo around day 12 post-fertilization and later differentiate to form the foundation for gametogenesis (Chen et al., 2019; Castillo-Venzor et al., 2023). After the initial period of specification and during embryonic development, PGCs undergo a maturation process during which dynamic changes in their transcriptome occur alongside major rearrangements of their epigenetic landscape (Murase et al., 2024; Guo et al., 2015).

The restricted accessibility to human embryos limits the study of *in vivo* hPGC differentiation and highlights the importance of IVG research. In the past decades, various protocols for human PGC-like cell (hPGCLC) specification have been successfully developed, but it remains unclear why hPGCLCs fail to fully mature (Irie et al., 2015; Overeem et al., 2023; Sasaki et al., 2015; Vijayakumar et al., 2023). To characterize the *bona fide* identity of hPGCLCs, most studies focus on the analysis of the transcriptome and methylome (Vaz Santos et al., 2025). Although the transcriptome provides us with valuable information about gene expression patterns, it does not offer a complete overview of the functional profile of the cell.

In the regulation of cellular function, metabolism plays a key role. Through its involvement in energy production, biosynthesis of essential macromolecules, and modulation of the epigenome, metabolism directly influences cellular differentiation and fate determination (Verdikt and Allard, 2021; Haws et al., 2020; Hayashi and Matsui, 2023; Tischler et al., 2019). Previous studies were able to show distinct metabolic features of mouse PGC (mPGC) in comparison to their pluripotent stem cell progenitors (embryonic stem cells), while reporting how metabolism directly regulates germ cell development (Hayashi et al., 2017a, 2024). It was shown that glycolysis decreases and oxidative phosphorylation (oxphos) increases along mPGC differentiation, with mouse PGCLCs (mPGCLCs) showing similar levels to those of the progenitors and early-stage mPGCs (E9.5). Furthermore, the inhibition of oxphos or glycolysis impaired early or late differentiation of mPGCLCs, respectively (Hayashi et al., 2017a). Similar trends of decreased glycolysis and increased OXPHOS were reported in hPGCs at weeks 4 and 9 by transcriptomics analysis in humans (Floros et al., 2018). Therefore, besides being essential for the development of working protocols for human IVG, other omics approaches, such as metabolomics and proteomics, are also required for mechanistic insights into the biology of hPGC formation. In addition to essential growth factors and a functional somatic niche (Lakshmipathi et al., 2025), both necessary to support continued germ cell development, it is possible that specific nutrients or oxygen/CO_2_ levels are also required to enable *in vitro* germ cell development beyond the PGC stage.

We performed metabolomics and proteomics assessments using mass spectrometry analysis to compare *in vitro* differentiated hPGCLCs, the non-hPGCLC fraction after differentiation and their progenitor human iPSCs. With this, we detected shifts in the canonical and noncanonical citric acid cycle tricarboxylic acid cycle (TCA), alongside glycolytic pathway remodeling. This includes isoform switches and downregulation of late-stage glycolysis proteins. Additionally, the pentose phosphate pathway (PPP) also revealed changes, with indications of a reduction in *de novo* nucleotide synthesis and an increase in nucleoside precursor production in hPGCLCs. By providing an integrative map of these metabolic networks, we intend to characterize the metabolic profile of early hPGCLCs to contribute to the development of improved *in vitro* culture systems of early human germ cells.

## RESULTS

### Distinct metabolic and proteomic profiles between the progenitor, hPGCLC and non-hPGCLC groups

To investigate the metabolic profile and the changes that occur during the early stage of hPGCLC *in vitro* differentiation, we performed metabolomic and proteomic analyses using ultrahigh-performance liquid chromatography-mass spectrometry. For this, three groups of cells were analyzed: (1) the iPSCs that served as the progenitors before *in vitro* differentiation; (2) the *in vitro*-derived hPGCLCs sorted using fluorescence-activated cell sorting (FACS) based on ITGA6+/EpCAM+; and (3) the remaining ITGA6^−^ non-hPGCLCs. The hPGCLCs and non-hPGCLCs were sorted on the fifth day of differentiation (Fig. S1). The differentiation of hPGCLCs was done using an established protocol during which the cells were also characterized at the transcriptomic level (Overeem et al., 2023). The success of differentiation from iPSCs was additionally confirmed for each individual experiment using immunofluorescence staining for the PGC markers OCT4 and BLIMP1 (Fig. S1). Principal component analysis (PCA) revealed that the metabolic and proteomic profiles of iPSCs were clearly distinct from those of differentiated cells (Fig. 1A). This suggests that most of the observed proteomic and metabolomic changes (Tables S1-S6) are associated with the general *in vitro* differentiation process of iPSCs, rather than specific differences between the differentiated hPGCLC and non-hPGCLC populations.

**Fig. 1.**
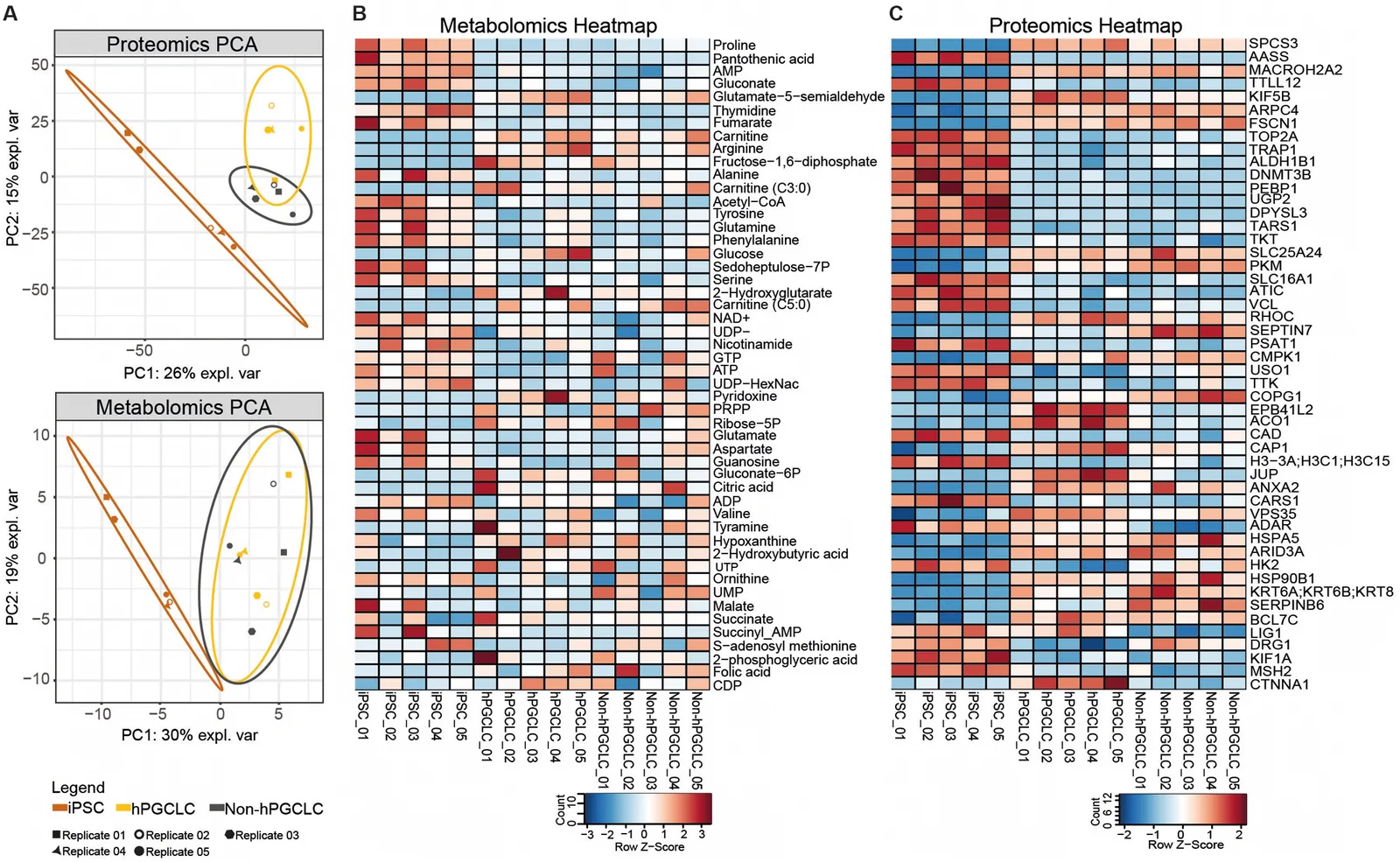
**Multicomparison of iPSC, hPGCLC and non-hPGCLC groups.** Five replicates were included per group for mass spectrometry analysis. (A) PCA of the proteome and metabolome of the three groups. Ellipses represent the 95% confidence regions for each group. (B) Heatmap of the top 50 metabolites and (C) proteins, based on *P*-value.

Regarding the proteome, all three groups clustered separately, with a clear separation between the progenitor cells (iPSCs) and differentiated cells (hPGCLCs and non-hPGCLCs) (Fig. 1A). For the metabolome, PCA showed that the hPGCLC and non-hPGCLC groups almost completely overlap, with only the progenitor cells grouping separately (Fig. 1A). The top 50 metabolites and proteins that most significantly contributed to the difference between all groups are shown in the heatmaps (Fig. 1B,C) and reflect the similarity between the differentiated groups (hPGCLC and non-hPGCLC) as well as the within-group variation at the metabolite level.

The proteome analysis for the differentiated cell groups, hPGCLCs and non-hPGCLCs, identified a total of 487 significantly differentially expressed proteins (Table S4). Alongside the top 50 most significant proteins (Fig. 1C), hPGCLCs showed upregulation of proteins associated with DNA repair mechanisms (LIG1, PARP1, ADAR), chromatin remodeling (CBX1, CBX3, RBBP4), cell-cell adhesion (CTNNA1, CTNNA2, JUP, CTNNB1), nucleotide synthesis (PRPS1, PRPS2) and membrane transporters (SLC3A2, SLC2A3). On the other hand, hPGCLCs showed downregulation of proteins associated with cell cycle progression and division (CDK1, MAD2L1, SEPTIN7), translation factors and protein modification (EIF3F, EIF2S1, EIF3L, UBE2D2, UBE2D3), glycolysis metabolism (LDHA, LDHB) and retinoic acid signaling (CRABP2, RBP1).

Functional enrichment analysis of the differentially regulated proteins identified several enriched terms (Tables S7-S12). In comparison to the differentiated non-hPGCLCs, the hPGCLCs revealed enriched terms associated with cell adhesion and cytoskeleton, cellular traffic and localization, and indole-3-methanol response (Fig. 2A). Simultaneously, the hPGCLCs showed a decrease of terms associated with nucleoside metabolism, sugar metabolism (namely, glycolysis/gluconeogenesis) and central carbon metabolism (Fig. 2A). In comparison with the progenitor iPSCs, the hPGCLCs showed enriched terms associated with cell adhesion and cytoskeleton and cellular and organelle organization (Fig. 2B). The downregulated terms were associated with translation, RNA binding and amino acid metabolism (Fig. 2B). However, the comparison between non-hPGCLCs and iPSCs revealed similar upregulated and downregulated terms as those observed when comparing iPSCs with hPGCLCs (Tables S7 and S8).

**Fig. 2.**
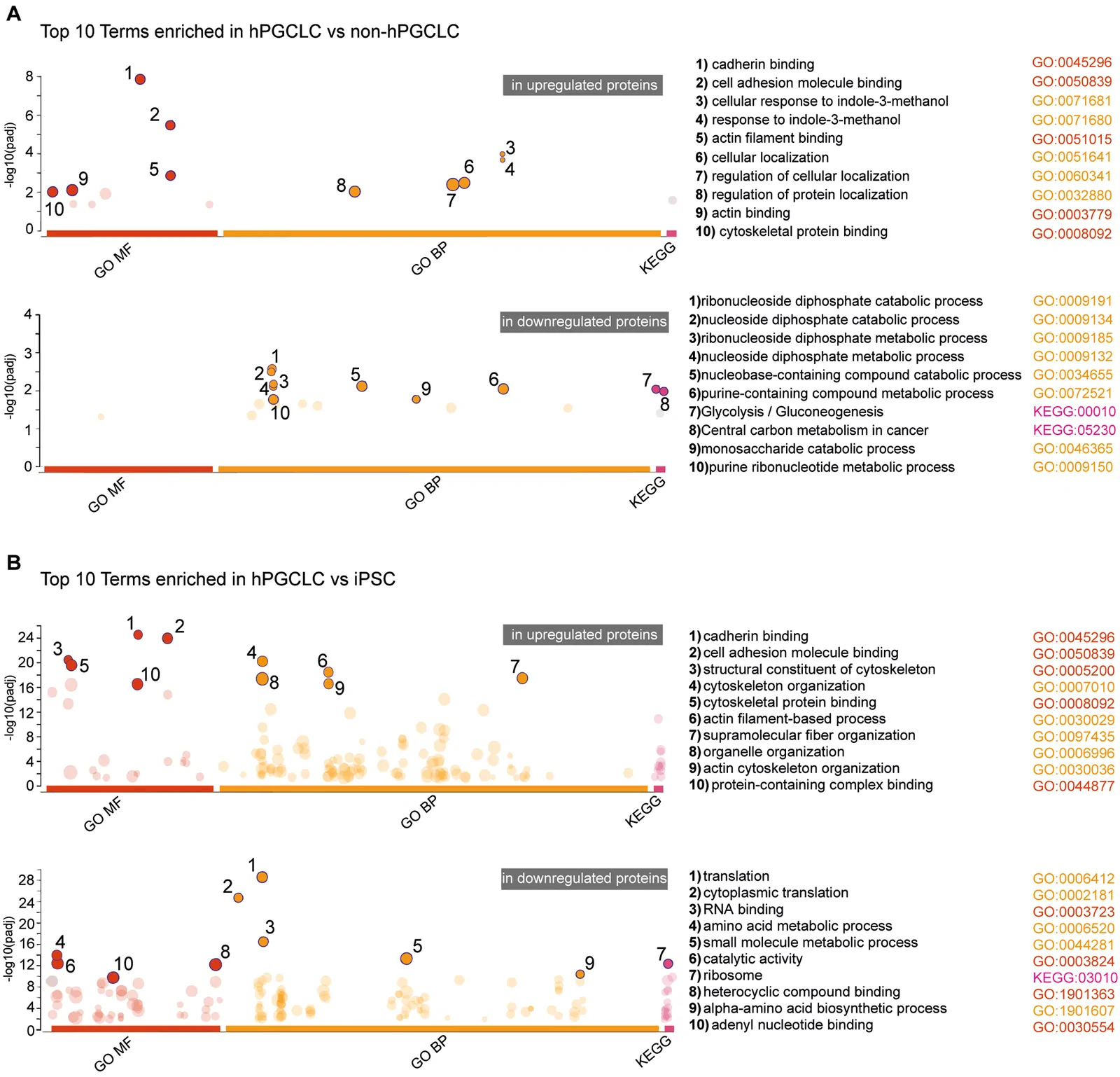
**Top ten enriched terms (*P*≤0.05, g:Profiler g:GOSt) of Gene Ontology (GO) analysis for significantly upregulated and downregulated proteins.** (A) hPGCLCs versus non-hPGCLCs and (B) hPGCLCs versus iPSCs. Enrichment for terms in categories GO Molecular Function (red), GO Biological Process (orange) and KEGG Pathways (magenta). Circles in transparent tone represent significantly enriched terms that are not part of the top ten; the size of the circle represents the number of proteins included.

### Energy metabolism of hPGCLCs is characterized by remodeling the glycolytic pathway and upregulating noncanonical TCA-associated proteins

Energy production in human cells relies mostly on two major pathways: glycolysis and oxphos. Oxphos is preceded by the TCA, where the electron carriers required for the function of the electron transport chain (ETC) are produced.

Compared to iPSCs for energy-associated metabolites, hPGCLCs exhibited increased levels of glucose and fructose-1,6-diphosphate and decreased levels of pantothenic acid, fumarate, ATP, AMP, and GTP (Fig. 3A). No significant differences in metabolite levels were observed between the two differentiated cell types; both hPGCLCs and non-hPGCLCs showed similar changes relative to iPSCs, except that non-hPGCLCs did not display downregulation of ATP or GTP (Fig. S3A).

**Fig. 3.**
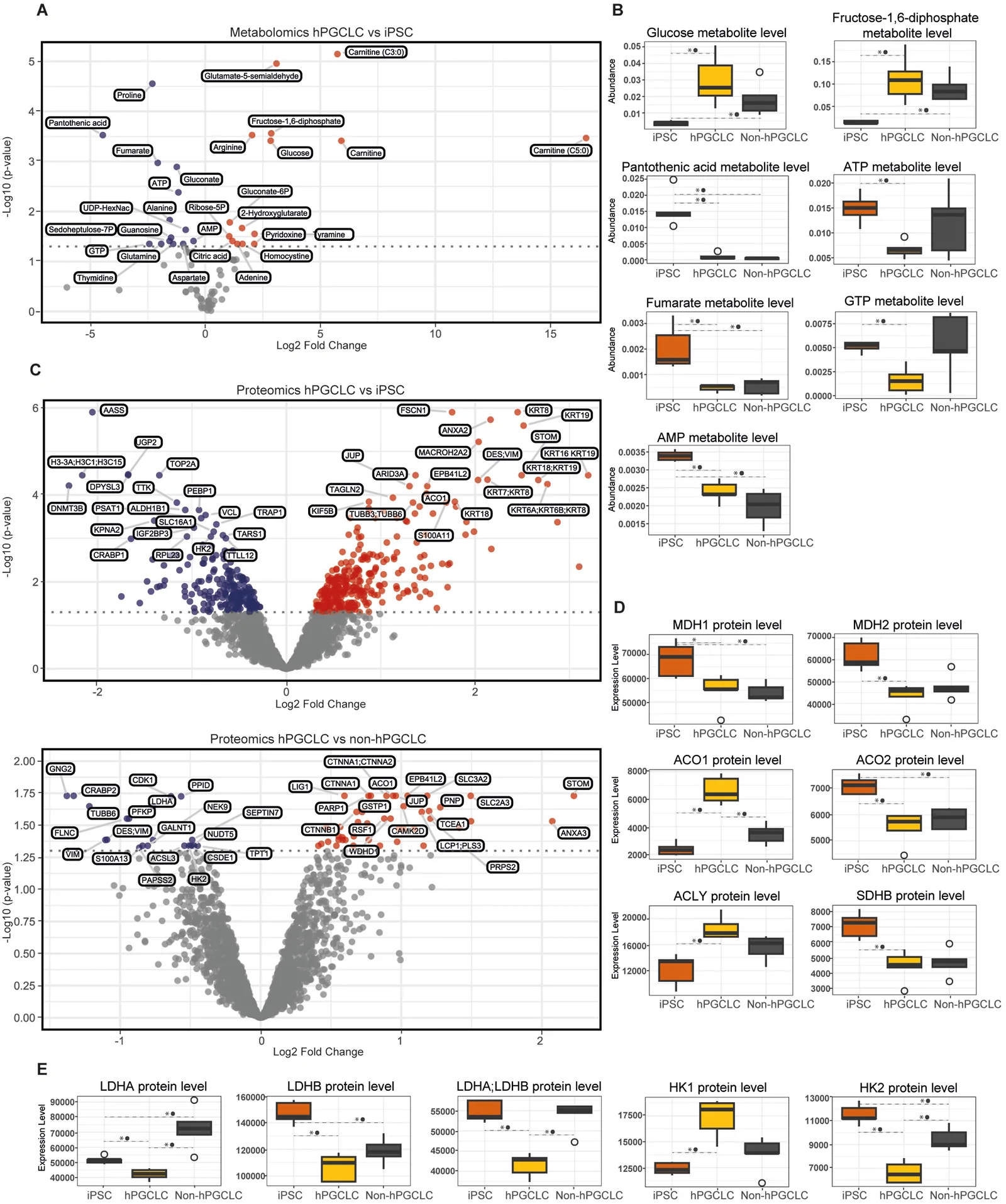
**Energy metabolism of hPGCLCs is characterized by remodeling the glycolytic pathway and upregulating noncanonical TCA-associated proteins.** (A) Volcano plot of the metabolome comparison between hPGCLCs and iPSCs; metabolites organized by *P*-value and increase in (red) or decrease in (blue) hPGCLC abundance. (B) Boxplot of metabolites related to glycolysis or oxphos. (C) Volcano plot of the proteome comparison between hPGCLCs and iPSCs and between non-hPGCLCs and hPGCLCs; proteins organized by *P*-value and increased (red) or decreased (blue) expression. (D) Boxplot of proteins related to oxphos and citric acid cycle or (E) glycolysis. * Five independent replicates. Upon empirical Bayes moderated *t*-test application, *P*≤0.05; • represents adjusted BH *P*≤0.05.

Overall, proteome analysis revealed broad changes between all three groups associated with energy-related pathways, with upregulation and downregulation of proteins in both the TCA cycle and glycolysis (Fig. 3C,D,E). For the TCA cycle, the hPGCLCs exhibited an upregulation of proteins constituting the non-canonical TCA pathway, namely ACLY, ACO1 (versus iPSCs and non-PGCLCs) and IDH1 (versus non-PGCLC). Of the canonical TCA cycle, both differentiated cell types showed downregulation of the TCA-associated proteins ACO2, SDHB, MDH1 and MDH2 in comparison to iPSCs (Fig. 3C). For the ETC, although some proteins showed differences between cell types, no general pathway change was observed (Tables S2 and S4).

The glycolytic pathway also presented significant changes, with hPGCLCs revealing downregulation of enzymes associated with late-stage glycolysis, LDHA/LDHB (versus iPSCs and non-hPGCLCs). Furthermore, a change in protein isoform levels was detected in hPGCLCs, with upregulation of HK1 to the detriment of HK2 (versus iPSCs and non-hPGCLCs) (Fig. 3E). To validate this result, an inhibitor assay was performed by adding 2-deoxy-D-glucose (2DG), a glycolytic inhibitor that targets the hexokinases and has been suggested to predominantly affect HK2 (Tseng et al., 2018; Wang et al., 2015; Tsai and Wilson, 1996), at days 2 and 3 onwards of differentiation. At day 4 of differentiation, flow cytometry analysis was performed to evaluate the percentage of live and dead cells in the hPGCLC and non-hPGCLC populations, both in the control condition without inhibitor and in the conditions with inhibitor. This showed that 2DG indeed preferentially kills non-hPGCLCs over hPGCLCs when added from day 2 (Fig. S4, Tables S13, S14, and S16).

An alternative energy source is through fatty acid oxidation. In this process, fatty acids are transported to the mitochondria, via the carnitine shuttle, for subsequent beta-oxidation, which will generate acetyl-CoA, NADH and FADH2 (de Carvalho and Caramujo, 2018). Both hPGCLCs and non-hPGCLCs presented a high increase in acylcarnitine metabolite levels in comparison to the progenitor cells (Fig. 3A; Fig. S2A).

### hPGCLCs reveal heterogeneous changes in the PPP and indicate reduction of *de novo* nucleotide synthesis

The metabolic reprogramming of the cell encompasses more than energy production. A parallel pathway to glycolysis, the PPP, directly regulates biosynthesis capacity. This occurs due to its capacity to oxidize glucose, derived from glycolysis, to produce ribose-5-phosphate and reduced nicotinamide adenine dinucleotide phosphate (NADPH), essential for redox balance and nucleotide synthesis, respectively (Kim et al., 2012).

At the metabolite level, compared to iPSCs, the hPGCLCs presented increased levels of ribose-5-phosphate and 6-phosphogluconate (activated form), while presenting decreased levels of gluconate (Fig. 4A).

**Fig. 4.**
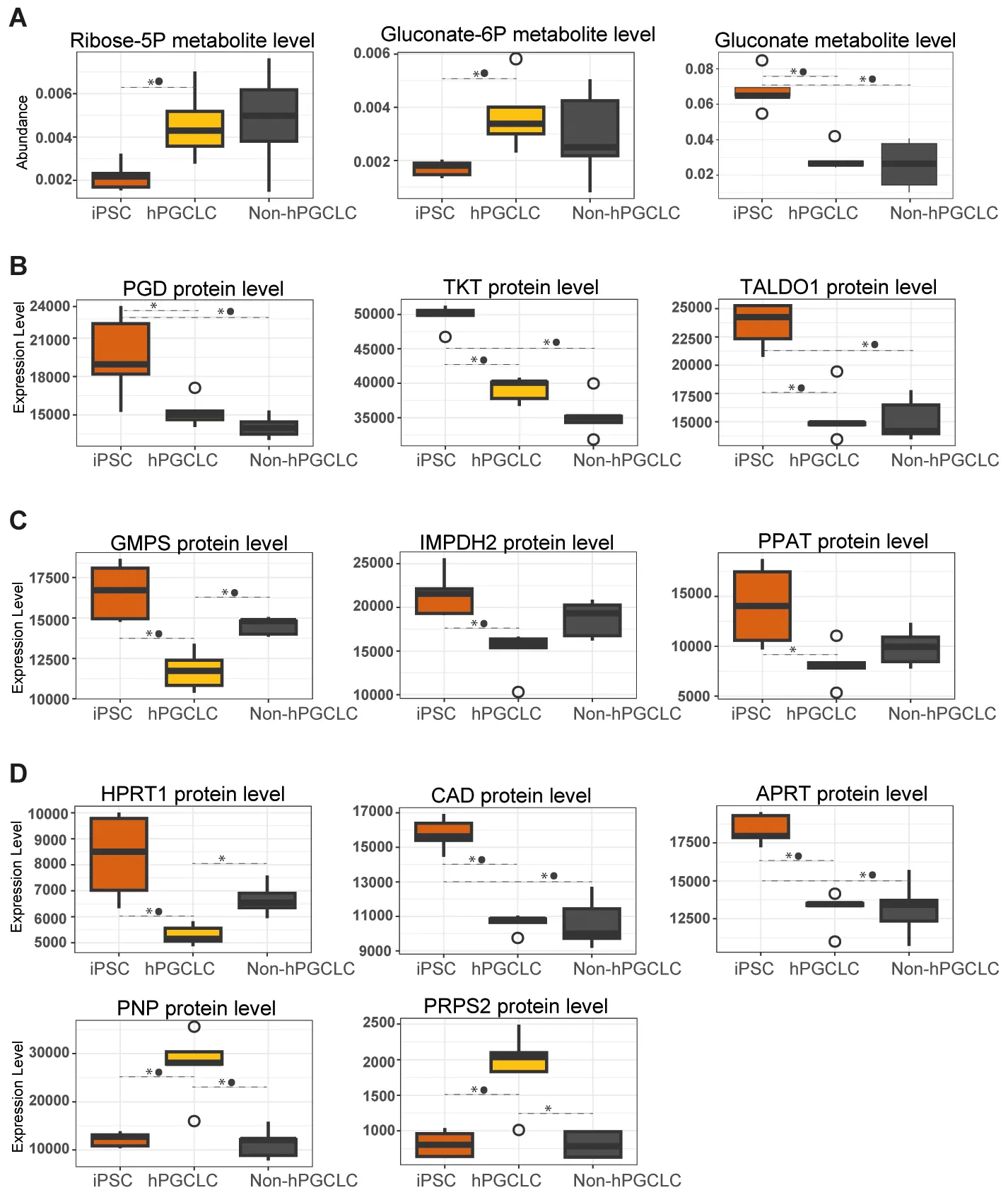
**hPGCLCs reveal heterogeneous changes in the PPP and indication of reduction of *de novo* nucleotide synthesis.** (A) Boxplot of metabolites involved in the PPP and nucleotide synthesis. Boxplot of proteins involved in (B) PPP, (C) *de novo* nucleotide synthesis and (D) purine nucleotide salvage and nucleoside recycling. * Five independent replicates. Upon empirical Bayes moderated *t*-test application, *P*≤0.05; • represents adjusted BH *P*≤0.05.

Similar to what was found for glycolysis and TCA pathways, multidirectional effects were detected in the PPP at the proteome level. As part of the oxidative PPP stage, the hPGCLCs presented downregulation of 6-phosphogluconate dehydrogenase (PGD) (versus iPSCs). As part of the non-oxidative stage of the pathway, the hPGCLCs presented upregulation of transketolase (TKT) in comparison with the non-hPGCLCs and downregulation in comparison to the iPSCs. The protein TALDO, also part of the non-oxidative PPP, was downregulated (versus iPSCs and non-PGCLCs) (Fig. 4B).

Nucleotide synthesis is largely influenced by PPP activity, which provides the necessary components for this biosynthetic process (Kim et al., 2012). Related to this process, the hPGCLCs presented downregulation of GMPS and IMPDH2 (versus iPSCs and non-hPGCLCs) and PPAT and CAD (versus iPSCs), which are proteins associated with *de novo* purine and pyrimidine syntheses (Fig. 4C). In addition, the hPGCLCs showed lower levels of HPRT1 (versus iPSCs and non-hPGCLCs) and APRT (versus iPSCs), related to purine salvage. Furthermore, the hPGCLCs presented upregulation of PRPS2 and PNP (versus iPSCs and non-hPGCLCs), proteins associated with general purine synthesis substrate production and nucleoside degradation and recycling, respectively (Fig. 4D).

## DISCUSSION

The goal of this study was to characterize the metabolome and proteome of *in vitro-*derived hPGCLCs and determine how they differ from their progenitor iPSC and parallelly differentiated non-hPGCLC counterparts to better understand the energy metabolism of human PGCs. Overall, the hPGCLCs presented heterogeneous changes at the protein level in the PPP pathway, in comparison with both the iPSC and non-hPGCLC groups. Similarly, the nucleotide synthesis pathways revealed broad changes, suggesting that hPGCLCs opt for an energy cost reduction of *de novo* synthesis while increasing nucleoside precursors.

The metabolite levels revealed a distinct profile between hPGCLCs and progenitor cells, demonstrating that the metabolome changes with differentiation. Notably, the metabolome of hPGCLCs showed no significant differences from the parallelly differentiated non-hPGCLCs. However, the metabolic state is not always fully captured by metabolite levels at a single time point, as this approach does not account for changes in metabolic flux (Srivastava et al., 2016). The metabolite level might not change, but the activity level, such as gain or loss of metabolites, might. In addition, some metabolites can be redirected differently. For example, glucose-6-phosphate can be directed to either the PPP or glycolysis (Jiang et al., 2022). Nonetheless, a relevant finding is the high increase in acylcarnitine metabolite levels in both differentiated cell types in comparison to the progenitor cells. This could indicate that the observed alterations in the metabolome are a general feature of iPSC differentiation when using the Overeem protocol (Overeem et al., 2023). The increase in acylcarnitine metabolites could be indicative of alterations in fatty acid oxidation; however, no other findings in our dataset support this (Longo et al., 2016; Stephens et al., 2007). Acylcarnitine metabolite increase can also be indicative of cell stress (Calabrese et al., 2006). As such, for the antioxidant effect, it could prove beneficial for the overall culture system to add acetyl-L-carnitine supplementation to the differentiation media.

The metabolic network not only depends on metabolites but also on the abundance of regulatory proteins. Contrary to the metabolome, proteomic analysis revealed distinct metabolic profiles among all studied cell groups, suggesting that proteomic changes are a specific feature of differentiation to either hPGCLCs or non-hPGCLCs. It remains unclear why these proteomic changes do not correspond to differences in the metabolome. One possible explanation is that changes in protein expression may not have had sufficient time to result in measurable differences in metabolite levels. Overall, the results revealed distinct proteomic profiles between the hPGCLCs, non-hPGCLCs and the progenitor cells (iPSCs). The functional enrichment analysis revealed that hPGCLCs also presented upregulation of terms associated with cytoskeleton remodeling and cell adhesion, in comparison to both iPSCs and non-hPGCLCs. This could indicate that already from an early stage, these cells start to mechanistically prepare for the migration to the gonadal ridge, observed at a later stage of their *in vivo* development (Mollgård et al., 2010). Furthermore, the results revealed a distinct energy profile between the cell groups, characterized by significant changes at the proteome level in the TCA cycle, glycolytic pathway and PPP.

In this study, the hPGCLCs exhibited upregulation of proteins constituting the the non-canonical TCA, while both hPGCLCs and non-hPGCLCs showed downregulation of proteins linked to the canonical TCA cycle. The shift between canonical and noncanonical TCA cycles has been previously described in multiple cell types (Mateska and Alexaki, 2022). In embryonic stem cells (ESCs), the TCA cycle shifts from canonical to noncanonical when the cells differentiate and exit pluripotency, and blocking the noncanonical TCA pathway was shown to impede pluripotency exit, showing its importance in general cell lineage specification (Arnold et al., 2022). The switch to the noncanonical pathway can redirect the TCA cycle to an anabolic purpose, increasing the biosynthetic capacity of the cell. Extensive epigenetic reprogramming is one of the main events that occur during both *in vivo* PGC and *in vitro* hPGCLC differentiation (Murase et al., 2024; Sugawa et al., 2015). It is known that the switch to the noncanonical pathway can lead to increased production of acetyl-CoA and regeneration of NAD+, which can influence epigenetic reprogramming through histone acetylation and promote glycolysis, respectively (Doan and Teitell, 2022; Arnold et al., 2022). While our data indicates upregulation of proteins constituent of the non-canonical TCA in hPGCLCs in comparison to both iPSCs and non-hPGCLCs, no increase in NAD+ or acetyl-CoA was observed. In addition, no alpha-ketoglutarate was detected in our analysis, which was previously described as a key metabolite connecting metabolism and epigenetic reprogramming in mouse germ cells (Xing et al., 2020; Tischler et al., 2019). Overall, the observed changes could indicate metabolic regulatory rewiring, leading to a preference for noncanonical TCA in hPGCLCs. Furthermore, the functional enrichment analysis identified glycolysis/gluconeogenesis as a top ten downregulated term in hPGCLCs in comparison to non-hPGCLCs. Together with the downregulation of proteins associated with late-stage glycolysis observed in comparison to both iPSCs and non-hPGCLCs, this could suggest a metabolic reprogramming that directs the hPGCLCs to a less proliferative, more biosynthesis-prone state.

In addition to the late-stage glycolysis, our data suggest further remodeling of the glycolytic pathway in hPGCLCs. We observed that these cells show a switch in isoform preference between the enzymes of the first-committing step in glycolysis, in comparison to both iPSCs and non-hPGCLCs, from HK2 (predominantly active in iPSCs) to HK1. Previous research, in mice, has associated HK2 prevalence with highly glycolytic cells, which favor proliferation, such as cancer or stem cells (Wolf et al., 2011). HK1, on the other hand, can act to promote the shift of glucose-6-phosphate from the mitochondria to the cytoplasm, with the potential for metabolic rerouting of this substrate between the glycolysis pathway and PPP (de Jesus et al., 2022). Furthermore, we showed that the addition of the glycolytic hexokinase inhibitor 2DG from day 2 of differentiation preferentially kills non-hPGCLCs over hPGCLCs. Although this inhibitor acts by inhibiting the hexokinases, some studies suggest that it predominantly affects the HK2 function (Tseng et al., 2018; Wang et al., 2015; Tsai and Wilson, 1996). This result corroborates the hPGCLCs’ isoform preference for HK1 over HK2 shown by the proteomic data, conferring on these cells higher resistance to the inhibitor action. Interestingly, the effect of the inhibitor was not significant when added from day 3. Previously published data showed that the hPGCLC specification from progenitors occurs between days 2 and 3 of the differentiation protocol, and the nascent hPGCLC population is established at day 3 (Overeem et al., 2023). This could suggest that the effect of the 2DG inhibitor is susceptible to the developmental timing of cell populations.

Based on these results, the efficiency of glycolytic inhibitors for use on hPGCLCs, such as 2DG (commonly used in assays like the Seahorse extracellular flux), should be evaluated.

The PPP contributes to nucleoside production, biosynthetic capacity and redox control of the cell, through production of ribose-5-phosphate and NADPH. For its function, this pathway requires the use of the substrate glucose-6-phosphate, produced in the early steps of the glycolytic pathway (Jiang et al., 2014). Our data suggest modulation of PPP activity in hPGCLCs rather than a general activation or suppression, as evidenced by alterations on both the oxidative and non-oxidative stages of this pathway. Particularly, the changes in PGD levels, observed by both hPGCLCs and non-hPGCLCs in comparison to iPSCs, could suggest modulation of the NADPH production rate to maintain redox homeostasis (Dubreuil et al., 2020; Ju et al., 2020). Combined with the observed increase in metabolite levels when compared with iPSCs, this could indicate an increase of the oxidative PPP flux and possible biosynthetic capacity, when compared to the progenitor cells (Jiang et al., 2014; Polat et al., 2021). However, since no changes in NADPH or glutathione were detected, which are central predictors of the antioxidant and redox capacity of the cell, there is no direct evidence for alterations in redox metabolism. When compared to their differentiated counterpart (non-hPGCLCs), the increased levels of TKT could suggest a higher metabolic flexibility of the hPGCLCs, through interconversion of glycolytic products and pentose phosphates (Kim et al., 2012; Chen et al., 2022).

Nucleotide synthesis depends on ribose-5-phosphate derived from the PPP (Jiang et al., 2014). The hPGCLCs presented downregulation of proteins associated with *de novo* synthesis, while showing upregulation of proteins related to nucleoside recycling and nucleotide substrate production, in comparison to the iPSCs and non-hPGCLCs. Furthermore, the functional enrichment analysis revealed a downregulation of terms associated with nucleoside metabolism in comparison to non-hPGCLCs. This pattern could indicate a shift in the germ cell metabolic function by maintaining the nucleotide pool through less energy-demanding mechanisms, a characteristic of less proliferative, specialized cells (Madsen et al., 2023; Lane and Fan, 2015). Such metabolic restraint would align with the suggested energy-quiescent profile of hPGCLCs, enabling them to allocate resources toward biosynthetic processes and genomic stability.

In humans, a previous study with transcriptome analysis has shown the upregulation of glycolytic and downregulation of oxphos-associated genes in hPGCs at weeks 4 and 9 (Floros et al., 2018), representing a later developmental time point in comparison to our hPGCLCs. However, a direct measurement of metabolite or protein levels was not reported. Similar research has been done regarding metabolism in mPGCs, through direct measurements of protein and metabolite levels. The metabolic profile of mPGCs revealed a transition from a glycolytic state (E9.5), similar to their ESC progenitors, to a mainly oxidative state (E13.5) (Hayashi et al., 2017a). While our hPGCLCs presented pinpoint pathway changes instead of overall pathway transitions, the observed alterations targeted several of the same pathways as in mice. Both the hPGCLCs and mPGCs demonstrated changes at the TCA level, particularly focused on alpha-ketoglutarate regulation in the mouse system (Hayashi et al., 2017a; Tischler et al., 2019) and more focused on the noncanonical TCA in our human system. A similar parallel can also be drawn at the glycolysis level, with the mPGCs shifting from a glycolytic precursor to a highly oxphos state (Hayashi et al., 2017a), while the protein changes in hPGCLCs suggest downregulation of late-stage glycolysis. Furthermore, the changes observed in this study at the nucleotide synthesis level also relate to what was previously described in mouse cells (Hayashi et al., 2017a), indicating that both models seem to tend toward an energy-efficient nucleotide pool maintenance. When looking at the *in vitro*-differentiated mPGCLCs, the energy metabolism of these cells was more similar to that of their progenitor cells rather than mPGCs. In addition, the extracellular acidification rate and oxygen consumption rate of mPGCLCs indicated lower oxphos and similar glycolytic activity levels in comparison to mPGCs, suggesting that these levels are altered at a later stage of differentiation (Hayashi et al., 2017a). Such findings correspond with the results shown here for hPGCLCs, with rearrangements of components of both the TCA and glycolysis pathways. Overall, this could indicate that the maturation status of germ cells is an important factor for their metabolic rewiring since mPGCLCs correlate to an earlier developmental stage than the mPGCs and closely resemble the energy profile of their ESC progenitors. In addition, this would align with the metabolic rewiring identified at the transcriptomic level in later-stage hPGCs (Floros et al., 2018). A limitation of our study lies in the absence of more mature hPGCLCs. For further understanding of the evolving metabolic profile in hPGCLCs, it would thus be helpful to include *in vitro*-differentiated samples that correspond to a more mature hPGCLC developmental stage, allowing for a better mapping of the metabolic development along human germ cell differentiation.

Overall, the data presented in this study indicate that *in vitro* differentiation of iPSCs to hPGCLCs is characterized by a metabolic regulatory rewiring, mainly at the protein level. This is reflected by a more quiescent cell state, with hPGCLC-specific modulation of specific pathways that include a preference for noncanonical TCA activity, downregulation of late-stage glycolysis regulators and specific protein isoform switches in energy-related pathways. However, although these changes were shown at the protein level, they did not translate to metabolome changes. This was previously also observed in mPGCLCs and suggests the importance of further maturation for complete metabolic rewiring to take place (Hayashi et al., 2017a). Nevertheless, the newly obtained knowledge provides a better understanding of the initial steps of the metabolic reprogramming during early human germ cell development.

## MATERIALS AND METHODS

### Human iPSC culture

The human iPSCs used in the mass spectrometry experiment were derived from the LUMC0054iCTRL03 male cell line, provided by the LUMC iPSC core facility (Overeem et al., 2023). These cells were cultured at 37°C with 5% CO_2_, using mTeSR Plus media from STEMCELL Technologies, and were maintained for a maximum of 35 passages. Every 5-7 days, the cells were passaged, with the use of ReLeSR (STEMCELL Technologies), to a Geltrex-coated (Thermo Fisher Scientific) well containing mTeSR Plus media, 1% penicillin-streptomycin (PenStrep) and 1% RevitaCell (Thermo Fisher Scientific). The day after passaging, the media was replaced to remove the RevitaCell. The media was refreshed every other day. Mycoplasma testing was performed monthly on cultured iPSCs. The iPSCs were kept in culture for no more than 15 passages, up to a maximum of 30 passages. Frozen iPSC stocks were stored in liquid nitrogen. RNA expression of pluripotency markers *Oct4*, *Nanog* and *Sox2* in undifferentiated iPSCs was confirmed by quantitative (q)PCR (Fig. S5). qPCR data were analyzed with RDML GEAR Tools v1.4 (Untergasser et al., 2026). The human iPSCs (585B1-868 male cell line, harboring the reporters BLIMP1-tdTomato and TFAP2C-EGFP) (Sasaki et al., 2015) used for the 2DG inhibitor assay were maintained under similar conditions as the previous cell line, with some distinctions. These cells were maintained in iMatrix-511-coated (REPROCELL) wells, with the basal media StemFit Basic04 Complete (SanBio) containing mTeSR Plus media and 1% PenStrep. Every 5-7 days, the cells were passaged with the use of ReLeSR (STEMCELL Technologies), and the basal media was supplemented with 1% RevitaCell for 24 h.

### hPGCLC differentiation and isolation

hPGCLCs were differentiated according to a previously published protocol (Overeem et al., 2023). At day 0, single-cell iPSCs were resuspended in media composed of mTeSR Plus (cell line LUMC0054iCTRL03) or StemFit Basic04 Complete (cell line 585B1-868), RevitaCell and 2% Geltrex. The cells were seeded on Geltrex-coated plates at a density of 60.00 cells/cm^2^. On day 1, the media was removed, and the cells were washed with RPMI 1640 (Thermo Fisher Scientific). Differentiation media A was added to the cells, composed of basal media aRB27 [containing RPMI 1640, 1× GlutaMAX (Thermo Fisher Scientific), 1× MEM Non-Essential Amino Acids (Thermo Fisher Scientific), 1:100 B27 (Thermo Fisher Scientific) and 1% PenStrep] with 10 ng/ml human BMP4 (R&D Systems) and 2% Geltrex. On day 2, differentiation media A was refreshed. On day 3, the media was exchanged, and differentiation media B was added to the cells, composed of basal media aRB27 with 10 ng/ml human BMP4, 10 ng/ml human LIF (PeproTech), 50 ng/ml human stem cell factor (SCF) (PeproTech) and 50 ng/ml human epidermal growth factor (EGF) (PeproTech). At day 4 of differentiation, the heterogeneous population was prepared for FACS or flow cytometry. For this, the differentiated cells were dissociated into a single-cell suspension upon incubation for 10 min with Accutase. After washing, the cells were incubated with a live-dead marker (for cell line LUMC0054iCTRL03, Zombie Green, BioLegend, cat. no. 423112; for cell line 585B1-868, eFluor 780, Invitrogen, 68-0865) at a concentration of 0.5 μL per million cells for 30 min at room temperature. The cells were then washed twice, for 5 min, with FACS buffer (PBS with 0.5% BSA). For samples derived from cell line 585B1-868, this was the final step, and the samples proceeded to be analyzed. For samples derived from cell line LUMC0054iCTRL03, the samples were incubated with the directly conjugated antibodies BV421-ITGA6 (BioLegend, cat. no. 313624) and APC-EpCAM (BioLegend, cat. no. 324208) at a concentration of 5 µl per million cells for 30 min at 4°C. After washing, the cell pellets were resuspended in FACS buffer, and the samples were analyzed by flow cytometry using a Sony SH800 sorter machine. For samples derived from cell line LUMC0054iCTRL03, both ITGA6+/EpCAM+ (hPGCLCs) and ITGA6−/EpCAM− (non-hPGCLCs) cell populations were sorted and collected for posterior analysis. In parallel, the progenitor iPSC population used to initiate the differentiation trial was also retrieved through dissociation of the colonies with Accutase and collection of the pellet. Upon collection of the cell samples, dry pellets corresponding to 250,000 cells per sample were snap frozen and stored at −80°C until further processing. For samples derived from cell line 585B1-868, data were collected by gating the populations for the live-dead marker and BLIMP1 and TFAP2C reporters. An unstained iPSC sample was used as a negative control for reporter expression.

### Mass spectrometry, sample processing and data analysis

A total of five replicates per group (iPSCs, hPGCLCs and non-hPGCLCs), collected from five independent differentiation trials, were included for mass spectrometry analysis. The iPSC samples of each replicate were collected at day 0 of the differentiation trial, from the same iPSC batch used for the differentiation trial. The metabolomics and proteomics unified extraction was performed, combining previously described protocols (Schomakers et al., 2022, 2025; Szyrwiel et al., 2024; Cox et al., 2014). For metabolomics, the following quantities of internal standard dissolved in water were added to each sample tube containing 250,000 cells in a dry pellet: adenosine-^15^N_5_-monophosphate (5 nmol), adenosine-^15^N_5_-triphosphate (5 nmol), alanine-^13^C_3_,^15^N (5 nmol), arginine-^13^C_6_ (5 nmol), aspartic acid-^13^C_4_ (5 nmol), D3-carnitine (0.5 nmol), D4-citric acid (0.5 nmol), ^13^C_1_-citrulline (0.5 nmol), cystine-^13^C_6_,^15^N_2_ (2.5 nmol), ^13^C_6_-fructose-1,6-diphosphate (1 nmol), glutamic acid-^13^C_5_ (5 nmol), D5-glutamine (0.5 nmol), glycine-^13^C_2_,^15^N (5 nmol), guanosine-^15^N_5_-monophosphate (5 nmol), guanosine-^15^N_5_-triphosphate (5 nmol), ^13^C_6_-glucose (10 nmol), ^13^C_6_-glucose-6-phosphate (1 nmol), histidine-^13^C_6_ (5 nmol), isoleucine-^13^C_6_,^15^N_5_ (5 nmol), D3-lactic acid (1 nmol), leucine-^13^C_6_,^15^N (5 nmol), lysine-^13^C_6_ (5 nmol), methionine-^13^C_5_,^15^N (5 nmol), D6-ornithine (0.5 nmol), phenyl-^13^C_6_-alanine (5 nmol), proline-^13^C_5_ (5 nmol), 1^3^C_3_-pyruvate (0.5 nmol), serine-^13^C_3_,^15^N (5 nmol), D6-succinic acid (0.5 nmol), threonine-^13^C_4_ (5 nmol), D4-thymine (1 nmol), tyrosine-(phenyl-^13^C_6_) (5 nmol), D5-tryptophan (0.5 nmol), valine-^13^C_5_ (5 nmol).

In the same tube, lipid-layer separation was performed (not included in this study). Afterward, solvents were added until a volume of 500 µl water and 500 µl methanol was reached. Sequentially, 1 ml of chloroform was added to each tube, and samples were properly mixed and then centrifuged at 14,000 rpm for 10 min to achieve layer separation.

The top layer, consisting of the polar phase, was transferred to a clean tube and dried using a vacuum concentrator. Posteriorly, the dried samples were reconstituted in 100 µl of 6:4 methanol/water. The metabolites were analyzed with a Waters ACQUITY UHPLC together with a Bruker Impact II™ ultrahigh-resolution Qq-time-of-flight mass spectrometer, and data were analyzed using Bruker TASQ software version 2.1.22.3. The chromatography column used for separation was a Merck Millipore SeQuant ZIC-cHILIC column, kept at 30°C. Mass spectrometry data were acquired using negative and positive ionization in full-scan mode over the range of m/z 50-1200 and analyzed using Bruker TASQ software version 2.1.22.3. Metabolite identification was based on a combination of accurate mass, (relative) retention times, ion mobility data and fragmentation spectra, compared to the analysis of a library of standards. Metabolites were normalized for the sum of all metabolites. No additional filtering, clustering or transformation was performed. After removal of the lipid layer, the protein pellet was freeze dried, and the sample preparation was done with a Thermo Fisher Scientific™ EasyPep™ MS Sample Prep Kit (A40006). Samples were reduced and alkylated at 95°C for 10 min, followed by a 2-h incubation at 37°C with a trypsin/Lys-C protease mixture. After sample cleanup using a peptide cleanup plate, samples were dried with nitrogen and sequentially resuspended in a 100 μl mixture of 97:3 (v/v) water:acetonitrile, containing 0.1% formic acid. Proteins were analyzed with a Waters™ ACQUITY UPLC BEH C18 chromatography column, kept at 60°C, paired with a Bruker timsTOF Pro 2 mass spectrometer using positive ionization in DIA-PASEF mode as previously reported (Szyrwiel et al., 2024). DIA-PASEF files were analyzed with DIA-NN version 1.8.1 (Demichev et al., 2020), using a spectral library provided by Bruker. Protein levels were normalized using the MaxLFQ method, and missing values were imputed using a K-nearest neighbor algorithm (Cox et al., 2014). For statistical analysis, differential protein and metabolite levels were assessed using the RStudio package Limma 3.64.3, and an empirical Bayes moderated *t*-test was applied, due to its capacity to stabilize variance estimates in small datasets. The *P*-values were adjusted using a Benjamini-Hochberg (BH) test to account for false discovery rates. Differences were deemed statistically significant when *P*≤0.05.

Functional enrichment analysis using GO was performed on the differentially expressed proteins, both upregulated and downregulated, separately, using g:Profiler g:GOSt (Kolberg et al., 2023). Protein identifiers were mapped with the background of all annotated human genes, with g:SCS multiple test correction and a threshold for significance of 0.05. Result terms were obtained for GO Biological Process, GO Molecular Function and KEGG Pathway.

### 2DG inhibitor assay and data analysis

For the 2DG inhibitor assay, the hPGCLCs were differentiated as previously mentioned. Four independent trials were performed, each consisting of three distinct conditions: control (normal differentiation without 2DG inhibitor), 2DG D2 (differentiation with 2DG inhibitor added from day 2 onwards) and 2DG D3 (differentiation with 2DG inhibitor added from day 3 onwards). Due to the metabolic role of the 2DG inhibitor (MedChemExpress, cat. no. HY-13966) that limits the use of toxicity assays, the inhibitor was added at a final concentration of 1 mM, according to previously published literature (Hayashi et al., 2017a,b). At day 4 of differentiation, the samples underwent FACS analysis.

FACS data were analyzed using FlowJo 10 software. To assess the 2DG inhibitor effect on cell death in both cell groups, the percentage of live cells was measured in both the hPGCLC (BLIMP1-tdTomato+/TFAP2C-EGFP+) and non-hPGCLC groups (single positive or negative for the reporters) (Table S13). Statistical analysis was performed in RStudio 2025.05.1+513. The percentages of live hPGCLCs and live non-hPGCLCs were normalized to the control of the corresponding trial to account for variation in differentiation efficiency between trials. Due to the limited replicate number and the skewed nature of ratios, the ratios were transformed to Log2 data to improve data symmetry optimal for statistical testing (Table S13). To calculate the difference in survival of non-hPGCLCs and hPGCLCs, we calculated, for each trial and condition, the differential Log2 ratio, corresponding to Log2 (live non-hPGCLC ratio) minus Log2 (live hPGCLC ratio) (Table S14). One-sample *t*-tests were performed to assess if the difference in survival of non-hPGCLCs and hPGCLCs, under different conditions, was significantly different from 0 [Log2 (ratio) equal to 0 corresponds to a ratio of 1]. To test if there was a difference in survival of non-hPGCLCs and hPGCLCs between inhibitor time points, we calculated the difference between the 2DG D2 and the 2DG D3 differential Log2 ratios (Table S15), and paired *t*-tests were performed on the results. The *P*-value was considered significant at ≤0.05 (Table S16).

### Immunofluorescence

For immunofluorescence staining, the cells were cultured in a Lab-Tek chamber slide with removable wells and fixed with 4% paraformaldehyde (PFA) for 10 min at day 4 of hPGCLC differentiation. After three washes with PBS, the cells were incubated with 0.5% Triton/PBS for 15 min at room temperature. Following three washes with 0.1% PBST, the cells were incubated with Superblock for 1 h at room temperature. Subsequently, the Superblock was removed, and the cells were incubated overnight at 4°C with the primary antibodies Oct3/4 (Santa Cruz Biotechnology, mouse sc-5279; 1:200) and Blimp1 (Invitrogen, rat 14-5963-82; 1:100), diluted in BrightDiluent. In parallel, for negative controls, one well was incubated with mouse IgG and rat IgG in the same concentrations as the primary antibodies. The following day, the cells were washed three times with 0.1% PBST. Next, they were incubated for 1 h at room temperature with the secondary antibodies anti-mouse Alexa Fluor 488 (1:1000) and anti-rat Alexa Fluor 555 (1:1000) and DAPI (1:1000). Lastly, the cells were washed three times with PBS and mounted with ProLong Gold.

### qPCR

Three distinct passages (p22, p23 and p27) of independent iPSC-cultured samples were used for this analysis. RNA was isolated with an RNeasy Plus Mini Kit according to the manufacturer's instructions (Qiagen, cat. no. 74134). cDNA was synthesized with an M-MLV reverse transcriptase (RT) kit (Promega, cat. no. M1705). MasterMix I contained, per sample, 1 µg RNA, 1 µg of 500 ng/µl random primers (Invitrogen, cat. no. 48190-011) and H_2_O, up to a total volume of 15 µl. After vortexing, the mixture was heated to 70°C for 5 min. MasterMix II contained, per sample, 5 µl of 5× first-strand buffer, 1.25 µl of 10 mM deoxyribonucleotide triphosphate (dNTP) mix (Thermo Fisher Scientific, cat. no. R0192), 0.625 µl RNasin 40 U/µl (Promega, cat. no. 2511), 1 µl of 200 U/µl M-MLV RT and H_2_O, up to a total volume of 10 µl. Upon the combination of 15 µl of MasterMix I with 10 µL of MasterMix II, the sample was incubated at 25°C for 10 min, followed by 1 h at 37°C and then cooled down to 4°C. Minus RT control was produced simultaneously. qPCR analysis was performed on a LightCycler 480 (Roche) using SYBR Green Mix (Roche, cat. no. 4887352001). Primers were used at a final concentration of 0.5 µM for target genes *Oct4* (Fw:AGGAA TCGGGCCGGGGGTTG/Rv:GTCCTGGGACTCCTCCGGGT; annealing temperature 68°C), *Nanog* (Fw:CCTGTGATTTGTGGGCCTGA/Rv:GGGTTGTTTGCCTTTGGGAC; annealing temperature 68°C) and *Sox2* (Fw:GTTACGCGCACATGAACGG/Rv:GTAGGACATGCTG TAGGTGGG; annealing temperature 65°C) and for reference genes *ActB* (Fw:CCTCGCC TTTGCCGATCC/Rv:CCACCATCACGCCCTGG; annealing temperature 62°C) and *Tmed1* (Fw:GGCCTGGAAGACCACCT/Rv:GCCACACCACCCTTTGTT; annealing temperature 62°C). In addition to three technical replicates (wells), minus RT and blank controls were included. qPCR data were analyzed with RDML GEAR Tools v1.4 (Untergasser et al., 2026).

## Supplementary Material



10.1242/biolopen.062801_sup1 Supplementary information

Table S1. Metabolite counts and statistical analysis for the comparison hPGCLC vs iPSC

Table S2. Protein counts and statistical analysis for the comparison hPGCLC vs iPSC

Table S3. Metabolite counts and statistical analysis for the comparison hPGCLC vs non-hPGCLC

Table S4. Protein counts and statistical analysis for the comparison hPGCLC vs non-hPGCLC

Table S5. Metabolite counts and statistical analysis for the comparison iPSC vs non-hPGCLC

Table S6. Protein counts and statistical analysis for the comparison iPSC vs non-hPGCLC

Table S7. Functional enrichment analysis results for upregulated proteins in comparison hPGCLC vs iPSC

Table S8. Functional enrichment analysis results for downregulated proteins in comparison hPGCLC vs iPSC

Table S9. Functional enrichment analysis results for upregulated proteins in comparison hPGCLC vs non-hPGCLC

Table S10. Functional enrichment analysis results for downregulated proteins in comparison hPGCLC vs non-hPGCLC

Table S11. Functional enrichment analysis results for upregulated proteins in comparison non-hPGCLC vs iPSC

Table S12. Functional enrichment analysis results for downregulated proteins in comparison non-hPGCLC vs iPSC
