# Supplementary material for: Characterization of the metabolome and proteome of stem cell-derived human primordial germ cell-like cells: a multi-omics approach
Source: Biol Open. 2026 Sep 18;15(9):bio062801. doi: 10.1242/bio.062801 (PMC13617983; doi:10.1242/bio.062801)
Supplement: Supplementary file 1 [file biolopen-15-062801-s1.pdf]

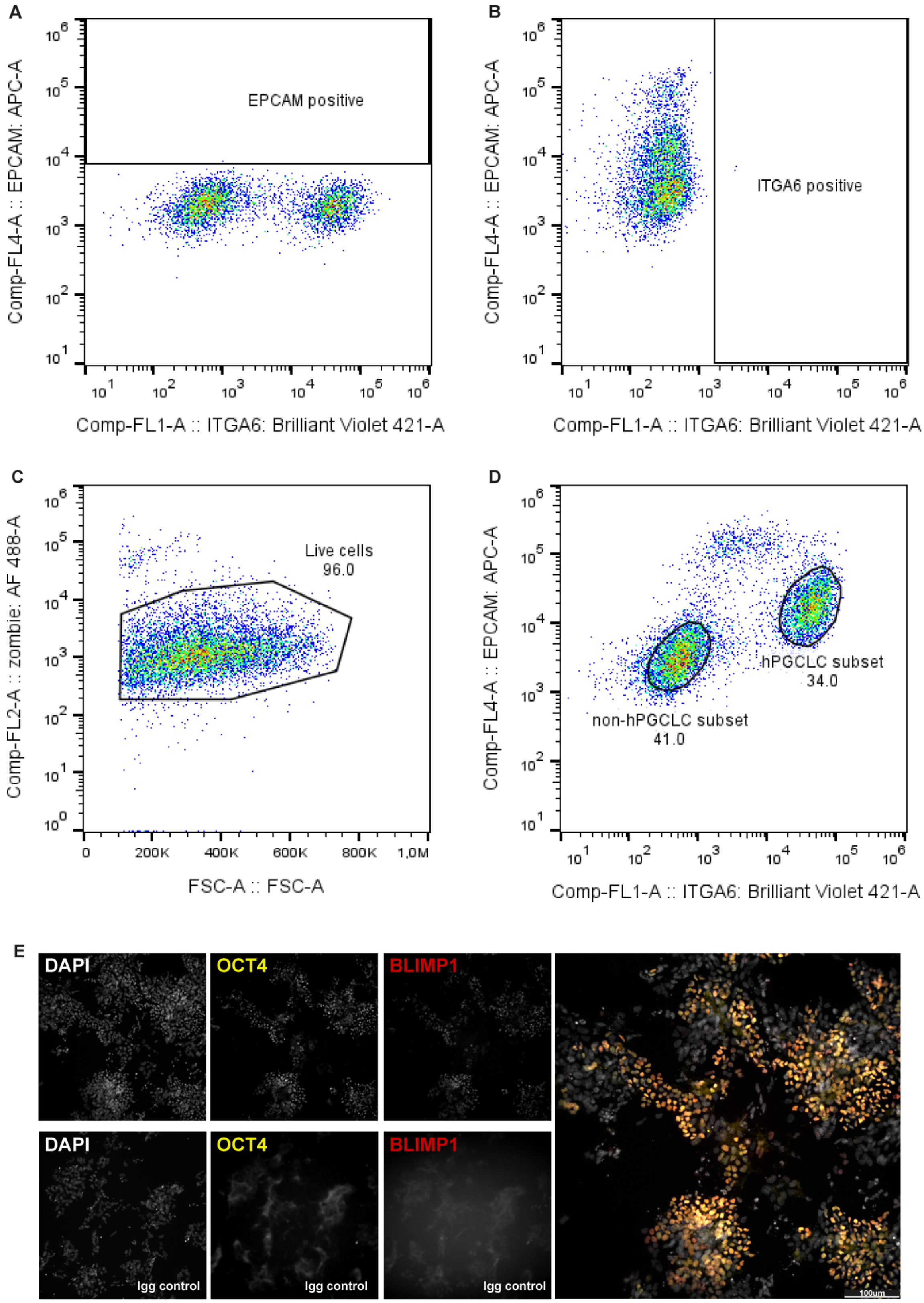

Biology Open • Supplementary information

Fig. S1. Validation of hPGCLC differentiation through immunofluorescence and Fluorescence-activated cell sorting of hPGCLC and non-hPGCLC groups, based on antibodies ITGA6 (biolegend, 313624) and EpCAM (biolegend, 324208) staining. **A**) FACS Fluorescence minus-one EpCAM control, **B**) FACS Fluorescence minus-one ITGA6 control, **C)** FACS Live cell population staining (Zombie AF 488) and selection, and **D)** FACS Gating of hPGCLC and Non-hPGCLC populations for sorting; **E)** Immunofluorescence staining of pre-sorting hPGCLC (and non-hPGCLC) population at day 4 of differentiation, for DAPI and primary antibodies Oct3/4 (santa cruz, sc-5279; 1:200) and Blimp1 (invitrogen, 14-5963-82; 1:100).

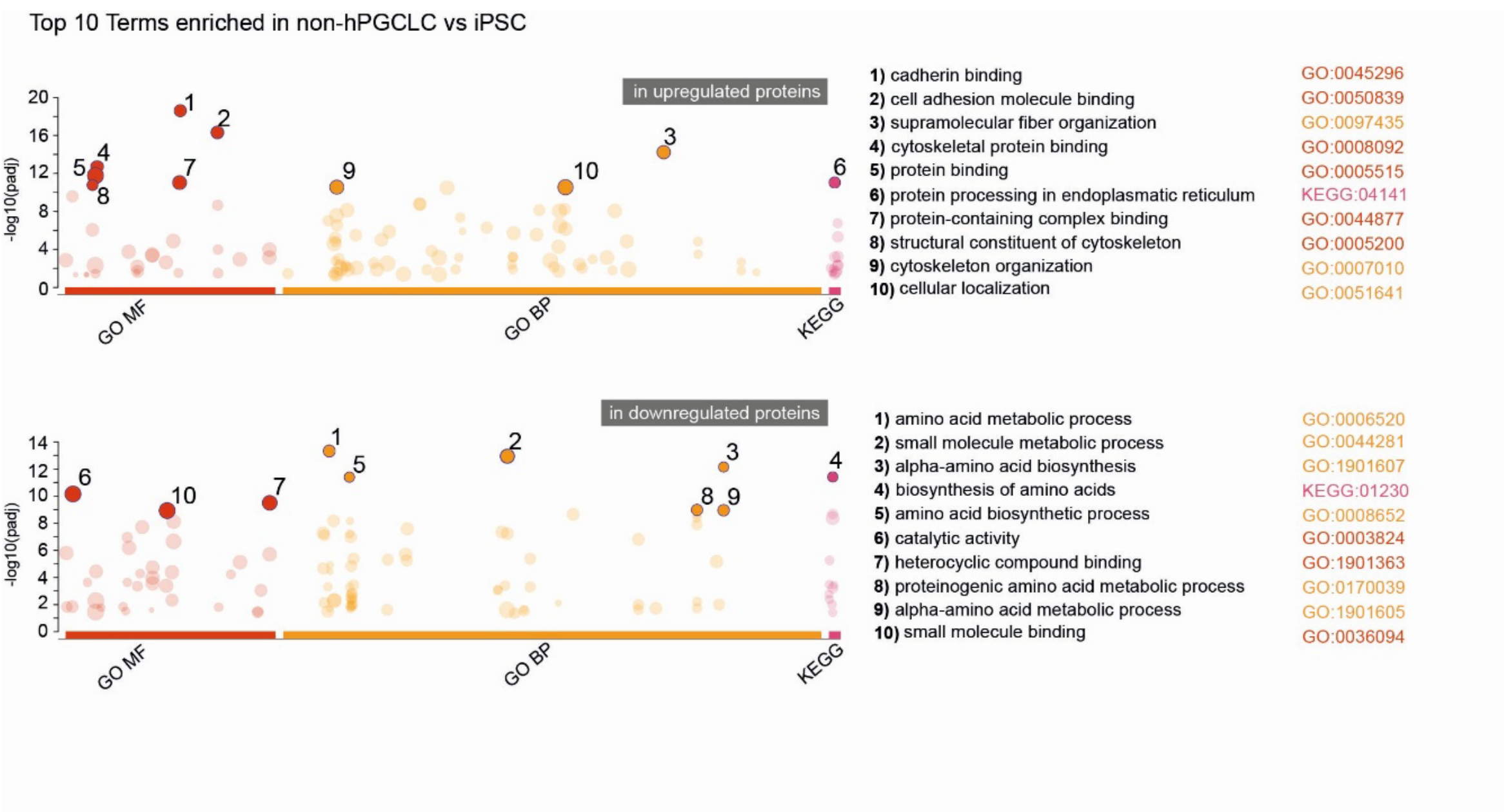

Fig. S2. Top 10 enriched terms (based on p-value) of gene ontology (GO) analysis for significantly up- and downregulated proteins in non-hPGCLCs vs iPSCs. Enrichment for terms in categories GO Molecular function (red), GO Biological process (orange) and KEGG pathways (magenta). Circles in transparent tone represent significantly enriched terms that are not part of the top 10; size of the circle represents number of proteins included.

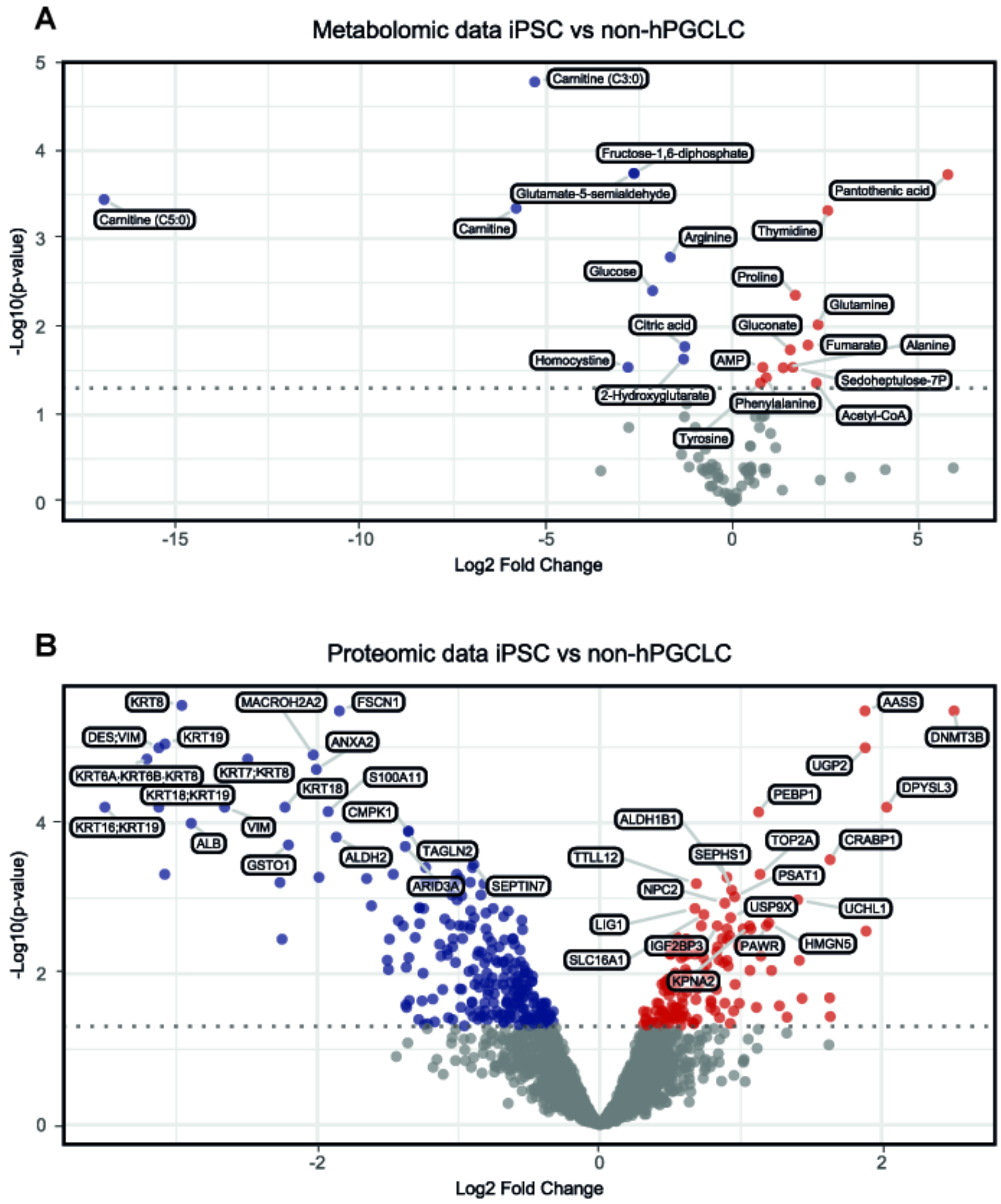

Fig. S3. Metabolome and proteome comparison of iPSCs vs non-hPGCLCs, based on data from Table S5 and S6. **A**) Volcano plot of the metabolome comparison; metabolites organized by p-value and increased (red) or decreased (blue) abundance; **B**) Volcano plot of the proteome comparison; proteins organized by p-value and increased (red) or decreased (blue) expression.

Biology Open • Supplementary information

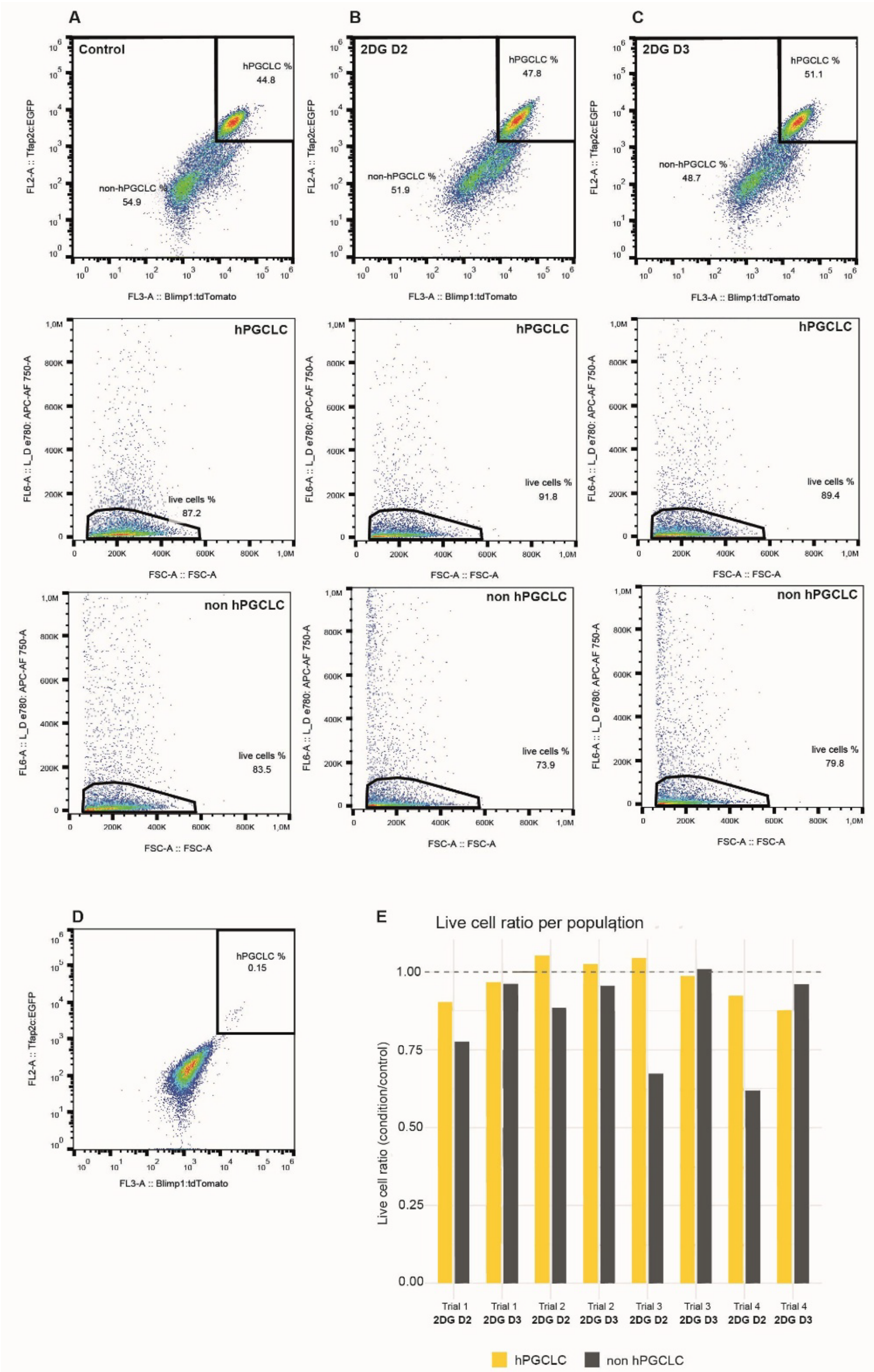

Fig. S4. Flow cytometry analysis of the percentage of live cells within the hPGCLC (positive for BLIMP1:tdTomato and TFAP2c:EGFP) and non-hPGCLC (negative for BLIMP1:tdTomato and TFAP2c:EGFP) populations, at day 4, after 2-Deoxy-D-glucose (2dg) inhibitor administration during hPGCLC differentiation. **A**) hPGCLC differentiation without 2DG addition, control condition; **B**) hPGCLC differentiation with 2DG addition from day 2 onwards; **C**) hPGCLC differentiation with 2DG addition from day 3 onwards; **D**) iPSC (cell line 585B1-868) unstained, negative control for reporter expression; **E**) Barplot of live cell ratio per cell population for each condition and trial. Live cell ratio's were calculated by dividing the percentage of live hPGCLCs or live non-hPGCLCs by the control of the correspondent trial, to account for variation of differentiation efficiency between trials.

Biology Open • Supplementary information

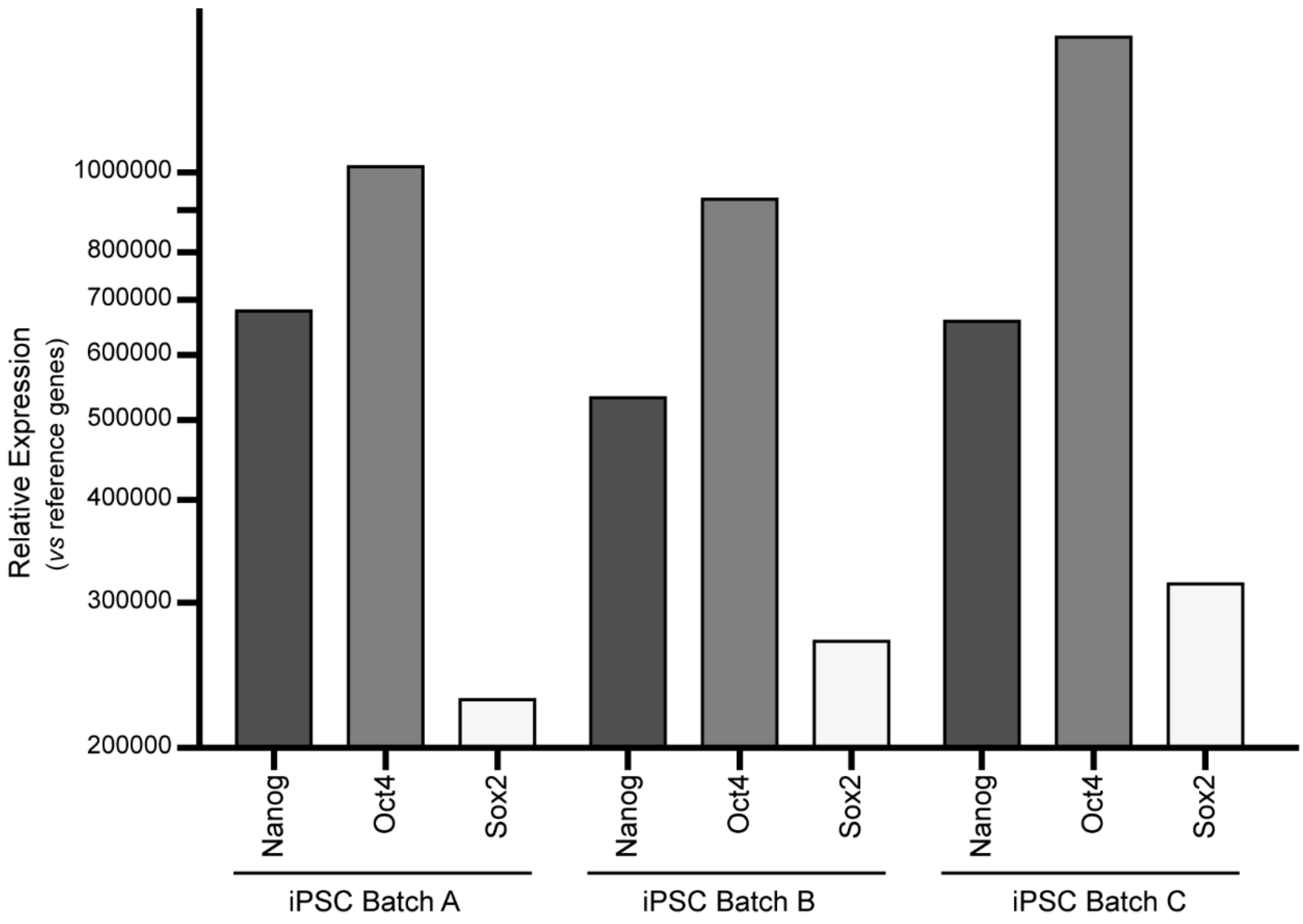

Fig. S5. Validation of pluripotency marker's RNA expression in undifferentiated iPSCs. Quantitative PCR performed for *Nanog*, *Oct4* and *Sox2*. Use of three independent iPSC cultured samples, correspondent to distinct passage numbers: passage 23 (iPSC Batch A), passage 27 (iPSC Batch B) and passage 22 (iPSC Batch C). Relative Expression is based on Ncopy values of the target genes normalized against reference genes. Each bar corresponds to the geometric mean of three technical replicates (wells).

Table S1. Metabolite counts and statistical analysis for the comparison hPGCLC vs iPSC

Available for download at
https://journals.biologists.com/bio/article-lookup/doi/10.1242/bio.062801#supplementary-data

Table S2. Protein counts and statistical analysis for the comparison hPGCLC vs iPSC

Available for download at

https://journals.biologists.com/bio/article-lookup/doi/10.1242/bio.062801#supplementary-data

Biology Open • Supplementary information

Table S3. Metabolite counts and statistical analysis for the comparison hPGCLC vs non-hPGCLC

Available for download at
https://journals.biologists.com/bio/article-lookup/doi/10.1242/bio.062801#supplementary-data

Table S4. Protein counts and statistical analysis for the comparison hPGCLC vs non-hPGCLC

Available for download at
https://journals.biologists.com/bio/article-lookup/doi/10.1242/bio.062801#supplementary-data

Table S5. Metabolite counts and statistical analysis for the comparison iPSC vs non-hPGCLC

Available for download at
https://journals.biologists.com/bio/article-lookup/doi/10.1242/bio.062801#supplementary-data

Table S6. Protein counts and statistical analysis for the comparison iPSC vs non-hPGCLC

Available for download at
https://journals.biologists.com/bio/article-lookup/doi/10.1242/bio.062801#supplementary-data

Table S7. Functional enrichment analysis results for upregulated proteins in comparison hPGCLC vs iPSC

Available for download at
https://journals.biologists.com/bio/article-lookup/doi/10.1242/bio.062801#supplementary-data

Table S8. Functional enrichment analysis results for downregulated proteins in comparison hPGCLC vs iPSC

Available for download at
https://journals.biologists.com/bio/article-lookup/doi/10.1242/bio.062801#supplementary-data

Table S9. Functional enrichment analysis results for upregulated proteins in comparison hPGCLC vs non-hPGCLC

Available for download at
https://journals.biologists.com/bio/article-lookup/doi/10.1242/bio.062801#supplementary-data

Table S10. Functional enrichment analysis results for downregulated proteins in comparison hPGCLC vs non-hPGCLC

Available for download at
https://journals.biologists.com/bio/article-lookup/doi/10.1242/bio.062801#supplementary-data

Table S11. Functional enrichment analysis results for upregulated proteins in comparison non-hPGCLC vs iPSC

Available for download at
https://journals.biologists.com/bio/article-lookup/doi/10.1242/bio.062801#supplementary-data

Table S12. Functional enrichment analysis results for downregulated proteins in comparison non-hPGCLC vs iPSC

Available for download at
https://journals.biologists.com/bio/article-lookup/doi/10.1242/bio.062801#supplementary-data

Table S13. Percentage of live cells and live cell ratio calculations, based on flow cytometry analysis, of hPGCLC and non-hPGCLC populations, for all trials and conditions of the 2-Deoxy-D-glucose (2DG) inhibitor essay.

Trial	Condition	Population	% live cells	Ratio live cells (vs CTR)
1	CTR	hPGCLC	81.9	-
1	CTR	Non hPGCLC	75.3	-
1	2DG D2	hPGCLC	74	0.9035
1	2DG D2	Non hPGCLC	58.5	0.7769
1	2DG D3	hPGCLC	79.2	0.9670
1	2DG D3	Non hPGCLC	72.4	0.9615
2	CTR	hPGCLC	87.2	-
2	CTR	Non hPGCLC	83.5	-
2	2DG D2	hPGCLC	91.8	1.0528
2	2DG D2	Non hPGCLC	73.9	0.8850
2	2DG D3	hPGCLC	89.4	1.0252
2	2DG D3	Non hPGCLC	79.8	0.9557
3	CTR	hPGCLC	73.4	-
3	CTR	Non hPGCLC	81.2	-
3	2DG D2	hPGCLC	76.7	1.0450
3	2DG D2	Non hPGCLC	54.7	0.6736
3	2DG D3	hPGCLC	72.5	0.9877
3	2DG D3	Non hPGCLC	81.9	1.0086
4	CTR	hPGCLC	79.2	-
4	CTR	Non hPGCLC	66.9	-
4	2DG D2	hPGCLC	73.2	0.9242
4	2DG D2	Non hPGCLC	41.5	0.6203
4	2DG D3	hPGCLC	69.5	0.8775
4	2DG D3	Non hPGCLC	64.3	0.9611

2DG: 2-Deoxy-D-glucose; D2: Day 2 of hPGCLC differentiation; D3: Day 3 of hPGCLC differentiation

Table S14. Log2 transformation of live cell ratios and differential Log2 (ratio of live cells) cal-culations of hPGCLC and non-hPGCLC populations, for all trials and conditions of the 2-De-oxy-D-glucose (2DG) inhibitor essay.

Trial	Condition	Live ratio_ hPGCLC (A)	Live ratio_ non hPGCLC (B)	Log2_live ratio A	Log2_live ratio B	Differential Log2 ratio*
1	2DG D2	0.9035	0.7769	-0.1463	-0.3642	-0.2179
1	2DG D3	0.967	0.9615	-0.0484	-0.0567	-0.0083
2	2DG D2	1.0528	0.885	0.0742	-0.1762	-0.2504
2	2DG D3	1.0252	0.9557	0.0359	-0.0654	-0.1013
3	2DG D2	1.045	0.6736	0.0634	-0.5699	-0.6334
3	2DG D3	0.9877	1.0086	-0.0178	0.0124	0.0302
4	2DG D2	0.9242	0.6203	-0.1137	-0.6889	-0.5752
4	2DG D3	0.8775	0.9611	-0.1885	-0.0572	0.1313
*Log2_live ratio A minus Log2_live ratio B						

2DG: 2-Deoxy-D-glucose; D2: Day 2 of hPGCLC differentiation; D3: Day 3 of hPGCLC differentiation

Table S15. Differential Log2 (ratio of live cells) calculations for statistical comparison of the 2-Deoxy-D-glucose (2DG) inhibitor effect between timepoints, for all trials

Trial	2DG D2_ Differential Log2 ratio	2DG D3_ Differential Log2 ratio	2DG D2_minus _2DG D3
1	-0.2179	-0.0083	-0.2096
2	-0.2504	-0.1013	-0.149
3	-0.6334	0.0302	-0.6636
4	-0.5752	0.1313	-0.7065

2DG: 2-Deoxy-D-glucose; D2: Day 2 of hPGCLC differentiation; D3: Day 3 of hPGCLC differentiation

Table S16. Statistical test results of differential Log2 (ratio of live cells), of hPGCLC and non hPGCLC populations, for all conditions of 2-Deoxy-D-glucose (2DG) inhibitor essay

Test	Mean_differential Log2 ratio	CI_lower	CI_upper	p_value
2DG D2: non hPGCLC vs hPGCLC live cells (differential Log2 ratio vs 0)	-0.4192	-0.762	-0.0763	0.0300
2DG D3: non hPGCLC vs hPGCLC live cells (differential Log2 ratio vs 0)	0.01296	-0.1402	0.1661	0.8051
2DG D2 vs 2DG D3 inhibitor effect (difference in differential Log2 ratio)	-0.4321	-0.8993	0.0349	0.0602

CI: 95% confidence interval for the mean; 2DG: 2-Deoxy-D-glucose; D2: Day 2 of hPGCLC differentiation; D3: Day 3 of hPGCLC differentiation
