# Supplementary material for: Characterization of the metabolome and proteome of stem cell-derived human primordial germ cell-like cells: a multi-omics approach
Source: Biol Open. 2026 Sep 18;15(9):bio062801. doi: 10.1242/bio.062801 (PMC13617983; doi:10.1242/bio.062801)
Supplement: Supplementary file 2 [file biolopen-15-062801_review_history.pdf]

Characterization of the metabolome and proteome of stem cell-derived human primordial germ cell-like cells: a multi-omics approach

Madalena Vaz Santos, Bauke Schomakers, Marta Llobet Ayala, Tara Jamali, Michel van Weeghel, Ans van Pelt, Callista Mulder and Geert Hamer
10.1242/bio.062801

Editor: Tristan Rodríguez

Review timeline

Original submission:	7 July 2026
Editorial decision:	15 July 2026
First revision received:	25 August 2026
Accepted:	26 August 2026

Original submission

<u>First decision letter</u>

MS ID#: bio.062801

MS Title: Characterization of the metabolome and proteome of stem cell-derived human primordial germ cell-like cells: a multi-omics approach

Authors: Madalena Vaz Santos; Bauke Schomakers; Marta Llobet Ayala; Tara Jamali; Michel van Weeghel; Ans van Pelt; Callista Mulder; Geert Hamer

I have now reached a decision on the above manuscript.

The reviewer reports are shown at the bottom of this email.

As you will see, the reviewers gave favourable reports, but raised some critical points that will require amendments to your manuscript. These primarily relate to providing additional information and clarifications. I do not think that there is a requirement for additional experimentation. However, should the requested experimental results be available then please provide these in the revised manuscript. I hope that you will be able to carry these out, because we would like to be able to accept your paper.

At this stage, we also ask you to ensure your manuscript complies with our formatting guidelines - please see our manuscript preparation guidelines for details. Provided you are able to fully address the referees' comments, we are positive about publication of your paper (we accept over 95% of revision submissions) and therefore hope you won't mind any extra work involved in reformatting your manuscript at this point.

Please upload both a 'clean' version of your Word file, along with a highlighted version clearly showing where you have made changes in the revised manuscript. Please avoid using 'Track changes' in Word files as these are lost in PDF conversion.

I should be grateful if you would also provide a point-by-point response detailing how you have dealt with the points raised by the reviewers in the 'Response to Reviewers' box. Please attend to

© 2026. Published by The Company of Biologists under the terms of the Creative Commons Attribution License (https://creativecommons.org/licenses/by/4.0/).

all of the reviewers' comments. If you do not agree with any of their criticisms or suggestions please explain clearly why this is so.

Reviewer 1

Comments for the author

This study by Vaz Santos et al., characterises the metabolome and proteome of newly derived hPGCLCs and compares them to those of IPSCs - the cells they were derived from - and non-hPGCLCs - the cells that failed to differentiate under the same conditions. While there is no comparison to mature PGCs or intermediate stages, this article produces a resource with potential to improve human stem cell differentiation.

1. Describe the derivation method used - perhaps as a diagram in Figure 1. This information is currently only available in the methods section as a reference to a paper.

2. Are non-hPGCLCs heterogeneous? In the paper where the derivation method was described, this population is described as containing both amniotic ectoderm and amniotic mesoderm cells. If that is the case, would it be worth having a more granular analysis between hPGCLCs and the different groups of non-hPGCLCs? Can the non-hPGCLCs not be separated based on their metabolome/ proteome?

3. The comparison between hPGCLCs and non-hPGCLCs is the most interesting one since they both grow in the same media and yet react differently to it. The metabolome/ proteome differences are not justified by the media they grow in but by intrinsic differences. This does not seem to be covered so much in the discussion. Additionally, it would be good to report in the text the average % of each population after the differentiation (differentiation success rate or mention Figure S1)

4. Why is there no mature PGC population in this analysis? Authors discussed that "it would be helpful to include in vitro differentiated samples that correspond to more mature hPGCLC developmental stage, allowing for a better mapping of the metabolic development along human germ cell differentiation" - why was this not the case? It should be mentioned as a limitation.

5. Why is there no intermediate population (< D4) being analysed to try and improve the differentiation? The study aims to complement the transcriptomic characterisation from Overeem et al., 2023 but in that paper; DO, D2 and D5 were analysed. This is especially notable because the discussion mentions that the metabolite differences might not be captured at a single time point. If it was not possible, it should be added as a limitation.

6. Authors mentioned that "a relevant finding is the high increase of acylcarnitine metabolites levels in both differentiated cell types in comparison to the progenitor cells. This could indicate that the observed alterations in the metabolome are a feature of differentiation in general from iPSC" (lines 214-216). Are there other studies to confirm this? Do you mean as a feature of general differentiation or differentiation into PGC progenitors? Please add a reference.

7. The results will be stronger with a different assay to confirm the findings, such as staining for some of the TCA enzymes (ACLY, ACO1) or another assay to measure glycolysis? Diverse assays would help to establish the potential heterogeneity of these results between cells.

8. Add more information about which statistical tests were performed to analyse the data in the methods and potentially also in the Figure legends.

Minor points:

- Mention the differentiation protocol in the paper, citing a paper in the Methods section is not sufficient. This could be added as a diagram in Figure 1.

- Figure 1, the 5 replicates - could they be indicated with shapes as to see if some of the differences within each population could be explained by the technical repeats?

© 2026. Published by The Company of Biologists under the terms of the Creative Commons Attribution License (https://creativecommons.org/licenses/by/4.0/).

- Also in Figure 1, this might be down to the quality of the PDF figure, but is there only 3 hPGCLC samples for the proteomics PCA?

- Also in Figure 1, what is the rationale for the circles surrounding each cell type? This is not explained in the paper, please add to the Figure legend.

- Figure 2, please add the comparison between IPSCs and non-hPGCLC in the figure

- Figure 3, why is there no metabolome analysis between hPGCLCs and non-hPGCLCs? Is it because there are barely any differences? Would it then be worth it to also add non-hPGCLCs into this graph?

- Line 259, additional space in HK 2 (HK2)

<u>Reviewer 2</u>

Comments for the author

The manuscript tackles an important and unaddressed question regarding in vitro differentiation of human primordial germ cells: how do proteins and metabolites change during the first steps of hPGCLC differentiation. For this they use an established 5-day 2D differentiation protocol to obtain hPGCLCs from hiPSCs and analyse the proteome and metabolome. This is a descriptive study in which the experimental design is well performed and uses a high number of replicates which should make the data robust and of high value for the scientific community. Despite the general interest of the data presented, some issues should be addressed to make it suitable for publication.

- There is little information in the Methods section or Figure Legend on how the data has been processed for Figure 1 in terms of missing values, filtering, normalization, clustering/transformation method used, etc. As such, it is difficult to interpret the results. The PCAs for both the proteome and the metabolome seem to suggest there is a batch effect for the iPSCs, so two iPSC clusters, of three and two samples respectively, are observed. Have the authors taken this into account? Or is there an alternative explanation for the distribution of iPSC samples into two groups? Related to this, it should be clarified in the Methods whether the iPSC sample for each experiment was collected the day the differentiation protocol started (day 0). Additionally, the proteome PCA seems to suggest there is an outlier in the hPGCLCs that clusters together with non-hPGCLCs, have the authors taken this into consideration? Regarding the heatmaps, they do not seem very informative with respect to the clustering of differentially abundant metabolites or proteins between conditions. Maybe the authors should re-consider the clustering method or represent the data in a different manner.

- From the GO analysis of the proteome (Figure 2) the main conclusion seems to be that hPGCLCs have a mayor cell adhesion/cytoskeleton remodelling compared to both iPSCs and non-hPGCLCs. This can also be observed when looking at the differentially expressed proteins in the volcano plot in Figure 3. However, the authors have not provided much discussion about this result. How does this correlate with previously published data, including transcriptomic data? Additionally, as a control for the proteome, the authors do not mention whether the main proteins that determine the hPGCLC fate become up-regulated in this cell type compared to iPCSCs/non-hPGCLCs. In fact, this type of analysis could be more informative than the immunostainings provided in Figure S1E or the qPCR in Figure S3.

- Regarding the protein changes, some metabolism-associated proteins come up as differentially expressed between h-PGCLCs and non-hPGCLCs. However, the fact that there are no significant differences in metabolites between h-PGCLCs and non-hPGCLCs seems to suggest both cell types are metabolically similar and therefore this feature is not a determinant of whether a differentiating cell acquires a PGC fate under this particular protocol. The authors argue that protein changes may precede metabolic changes, which could only be tested by assessing metabolism at later stages. However, an alternative explanation may be that the observed protein changes are not sufficient to impact metabolism. In this regard, it is noticeable that non of the

© 2026. Published by The Company of Biologists under the terms of the Creative Commons Attribution License (https://creativecommons.org/licenses/by/4.0/).

proteome or metabolome results have been validated (by western blot/specific metabolic assays) in additional independent experiments.

- Finally, I find the discussion somewhat confusing as it does not clearly distinguish between hPGCLCs specific features and more general differentiation features (i.e. those that also affect non-hPGCLCs) and so it's not clear in what way their results could contribute to the improvement of hPGCLCs production.

Reviewer's Responses to Questions

Experimental quality
Are experiments performed using appropriate methods that will answer the question (or test the hypothesis or support the observations) posed by the authors? Is the right tool used for the job?
If 'No', please indicate reasons in Comments for Author box below.
Reviewer #1:
- Yes

Reviewer #2:
- Yes

Were the data analyzed using appropriate statistical tests?
If 'No', please indicate reasons in Comments for Author box below.
Reviewer #1:
- No

Reviewer #2:
- Yes

Reproducibility
Were experiments performed using adequate number of biological replicates?
If 'No', please indicate reasons in Comments for Author box below.
Reviewer #1:
- Yes

Reviewer #2:
- Yes

Is there sufficient raw data to assess the rigor of the analysis?
If 'No', please indicate reasons in Comments for Author box below.
Reviewer #1:
- Yes

Reviewer #2:
- Yes

Does the methods section provide sufficient detail to permit reproducibility?
If 'No', please indicate reasons in Comments for Author box below.
Reviewer #1:
- No

Reviewer #2:
- No

Completeness

© 2026. Published by The Company of Biologists under the terms of the Creative Commons Attribution License (https://creativecommons.org/licenses/by/4.0/).

Are the manuscript's conclusions supported by the data?
If 'No', please indicate reasons in Comments for Author box below.
Reviewer #1:
- Yes

Reviewer #2:
- Yes

Scholarship
Do the authors cite and discuss the merits of data that would argue for and against their conclusion?
If 'No', please indicate reasons in Comments for Author box below.
Reviewer #1:
- Yes

Reviewer #2:
- Yes

Does the manuscript title & abstract accurately reflect the contents of the manuscript, without hyperbole?
If 'No', please indicate reasons in Comments for Author box below.
Reviewer #1:
- Yes

Reviewer #2:
- Yes

First revision

Author response to reviewers' comments

This letter accompanies the submission of the revision of our manuscript, now entitled "*Characterization of the metabolome and proteome of stem cell-derived human primordial germ cell-like cells: a multi-omics approach*". We thank the editorial board and reviewers for their time and efforts put in our manuscript and their constructive suggestions.

The revisions we made based on these suggestions are highlighted in yellow in the revised version. A clean version with all changes accepted has also been submitted.

Below you can find our point-by-point answer to all the reviewers' comments.

All authors approved the revised manuscript and this submission. We thank you for your time and consideration and look forward to your response.

Reviewer 1

Comment: *"Describe the derivation method used - perhaps as a diagram in Figure 1. This information is currently only available in the methods section as a reference to a paper."*

Answer: We thank the reviewer for the time and effort put in our manuscript. Because the hPGCLC derivation method is simply an employment of a previously published protocol (Overeem et al., 2023) without any adaptations, we find it should not be highlighted with its own figure as if we developed the culture method. The original article, which is solely dedicated to this method, is open access and contains an excellent figure and in depth explanation. To provide more technical details, we have introduced more information regarding the derivation method in the materials and

© 2026. Published by The Company of Biologists under the terms of the Creative Commons Attribution License (https://creativecommons.org/licenses/by/4.0/).

methods section (lines 395-406).

Comment: *“Are non-hPGCLCs heterogeneous? In the paper where the derivation method was described, this population is described as containing both amniotic ectoderm and amniotic mesoderm cells. If that is the case, would it be worth having a more granular analysis between hPGCLCs and the different groups of non-hPGCLCs? Can the non-hPGCLCs not be separated based on their metabolome/ proteome?”*

Answer: The non-hPGCLCs are indeed a heterogeneous population, as described in the original article (Overeem et al., 2023). In our research, we did not focus on an in-depth characterization of the non-hPGCLC group. However, since all the replicates from this group cluster together in the PCA graphs, this suggests that all non-hPGCLC sorted cells represent a similar proteome. Regarding the metabolome, the non-hPGCLC profile can not be distinguished from the hPGCLC group, but the replicates still form one cluster.

Comment: *“The comparison between hPGCLCs and non-hPGCLCs is the most interesting one since they both grow in the same media and yet react differently to it. The metabolome/ proteome differences are not justified by the media they grow in but by intrinsic differences. This does not seem to be covered so much in the discussion. Additionally, it would be good to report in the text the average % of each population after the differentiation (differentiation success rate or mention Figure S1)”*

Answer: Clear reference to Figure S1, in which these percentages are indicated, has now been added to the text (line 106).

Comment: *“Why is there no mature PGC population in this analysis? Authors discussed that "it would be helpful to include in vitro differentiated samples that correspond to more mature hPGCLC developmental stage, allowing for a better mapping of the metabolic development along human germ cell differentiation" - why was this not the case? It should be mentioned as a limitation.”*

Answer: The culture method does not support further PGC maturation. For this study, we did not focus on developing a maturation protocol for the maturation of hPGCLCs. The goal was to understand what differences, if any, existed at this early stage in the metabolome and proteome of the hPGCLCs, in comparison to the other derived cell groups. However, the results obtained indeed revealed that the use of a more mature hPGCLC population could be useful to further understand the metabolic rewiring of these cells. As suggested, we have included a mention to this as a limitation (lines 354-358).

Comment: *“Why is there no intermediate population (< D4) being analysed to try and improve the differentiation? The study aims to complement the transcriptomic characterisation from Overeem et al., 2023 but in that paper; DO, D2 and D5 were analysed. This is especially notable because the discussion mentions that the metabolite differences might not be captured at a single time point. If it was not possible, it should be added as a limitation.”*

Answer: Indeed, in our study only the D0 (iPSC) and D4 timepoints were considered. Overeem et al 2023 mention that the nascent hPGCLC population is established at day 3. If an intermediary timepoint were to be added to assess the transition of hPGCLC metabolome and proteome profile, it would need to be at day 3. However, due to budget and technical limitations regarding the amount of material required for the mass-spectrometry analysis, we have decided to focus on the D4 (and D0) timepoint.

Comment: *“Authors mentioned that "a relevant finding is the high increase of acylcarnitine metabolites levels in both differentiated cell types in comparison to the progenitor cells. This*

© 2026. Published by The Company of Biologists under the terms of the Creative Commons Attribution License (https://creativecommons.org/licenses/by/4.0/).

could indicate that the observed alterations in the metabolome are a feature of differentiation in general from iPSC" (lines 214-216). Are there other studies to confirm this? Do you mean as a feature of general differentiation or differentiation into PGC progenitors? Please add a reference."

Answer: The quoted line refers to a feature of general differentiation from iPSCs, not particularly to hPGCLCs. This is due to the fact that both hPGCLCs and non-hPGCLCs showed a significant increase of acylcarnitines in comparison to the iPSCs, suggesting that this is not a specific feature of the hPGCLCs but rather a common feature of both groups resultant from the differentiation protocol itself. There are no other studies to confirm this because we use a very specific differentiation protocol. We clarified this in the revised Discussion (lines 228,229).

Comment: *"The results will be stronger with a different assay to confirm the findings, such as staining for some of the TCA enzymes (ACLY, ACO1) or another assay to measure glycolysis? Diverse assays would help to establish the potential heterogeneity of these results between cells."*

Answer: Even though we have focussed on mass spectrometry, we also had planned to include a Seahorse essay to include glycolytic activity measurements. However, due to the difficulty of culturing our hPGCLCs post-sorting for at least 48h, we were not able to proceed with this. However, as validation of our -omics data, we have added a 2DG inhibitor essay in combination with an iPSC human cell line with reporters for BLIMP1:tdTomato and TFAP2c:EGFP. For this experiment, we have added 2-Deoxy-D-glucose (2DG), a glycolytic hexokinase inhibitor, during differentiation of hPGCLCs, either from day 2 onwards or from day 3 onwards. A control differentiation without 2DG was also included. At day 4 of differentiation, we assessed the samples with flow cytometry and measured the percentage of live hPGCLCs (double positive for the reporters) and live non-hPGCLCs (not double positive for the reporters), separately in each of the conditions. We compared each condition to the control differentiation of the corresponding trial.

2DG has been reported to have a higher effect on HK2, the isoform preferred by non-hPGCLCs (according to our proteomic data). On the other hand, the hPGCLCs show a preference for isoform HK1 over HK2. Therefore, we would expect that the 2DG inhibitor would have a higher impact in the cell death of non-hPGCLCs in comparison to the hPGCLCs. Indeed, the results from the inhibitor essay show that addition of 2DG from day 2 onwards of differentiation preferentially kills non-hPGCLCs over hPGCLCs. This information has been added to the manuscript (lines 169-177, 283-293; Tables S13-16; Figure S4) that aligns with the HK1/HK2 findings of the proteomic data (lines 168-170;.

Comment: *"Add more information about which statistical tests were performed to analyse the data in the methods and potentially also in the Figure legends."*

Answer: Additional information regarding statistical analysis of the data was added to the text (lines 463-464, 474-480) and to the legend of Figure 1 (line 735-737).

<u>Minor points</u>:

Comment: *"Mention the differentiation protocol in the paper, citing a paper in the Methods section is not sufficient. This could be added as a diagram in Figure 1."*

Answer: We have introduced more detailed information regarding the derivation method in the materials and methods section and moved relevant information from supplemental information to the main manuscript.(lines 394-405). Also see our answer to the first comment.

Comment: *"Figure 1, the 5 replicates - could they be indicated with shapes as to see if some of the differences within each population could be explained by the technical repeats?"*

© 2026. Published by The Company of Biologists under the terms of the Creative Commons Attribution License (https://creativecommons.org/licenses/by/4.0/).

Answer: We have adjusted Figure 1 as requested. Indeed this adaptation separates the technical repeats without complicating the figure.

Comment: *"Also in Figure 1, this might be down to the quality of the PDF figure, but is there only 3 hPGCLC samples for the proteomics PCA?"*

Answer: There are indeed 5 hPGCLCs samples, represented by 5 yellow symbols, of which some slightly overlap. In the original figure these are all clearly visible. If reviewers are able to also open the submitted figures, besides the pdf conversion, you could confirm this. The different revised shapes also further separate the 5 samples in each group.

Comment: *"Also in Figure 1, what is the rationale for the circles surrounding each cell type? This is not explained in the paper, please add to the Figure legend."*

Answer: The ellipses (circles) represent the 95% confidence regions for each group. This information has now also been added to the figure legend.

Comment: *"Figure 2, please add the comparison between IPSCs and non-hPGCLC in the figure."*

Answer: Although we recognize the importance of the non-hPGCLCs vs iPSC comparison as complementary data, the center of our study focus is on characterization of the hPGCLCs. For this reason, we opted to add figures regarding non-hPGCLCs vs iPSC comparison to the supplements and refer to them when needed. We have therefore now added a new figure in the supplements, figure S2, to show the functional enrichment data of non-hPGCLC vs iPSC, as requested.

Comment: *"Figure 3, why is there no metabolome analysis between hPGCLCs and non- hPGCLCs? Is it because there are barely any differences? Would it then be worth it to also add non-hPGCLCs into this graph?"*

Answer: Indeed, in figure 3 the metabolome plot between hPGCLCs and non-hPGCLCs was not included because, as mentioned in the text, there were no metabolites with significant differences. The plots regarding non-hPGCLCs vs iPSC comparison can be seen in the supplements in figure S3.

Comment: "Line 259, additional space in HK 2 (HK2)"

Answer: The extra space has been removed.

Reviewer 2:

Comment: *"There is little information in the Methods section or Figure Legend on how the data has been processed for Figure 1 in terms of missing values, filtering, normalization, clustering/transformation method used, etc. As such, it is difficult to interpret the results. The PCAs for both the proteome and the metabolome seem to suggest there is a batch effect for the iPSCs, so two iPSC clusters, of three and two samples respectively, are observed. Have the authors taken this into account? Or is there an alternative explanation for the distribution of iPSC samples into two groups? Related to this, it should be clarified in the Methods whether the iPSC sample for each experiment was collected the day the differentiation protocol started (day 0). Additionally, the proteome PCA seems to suggest there is an outlier in the hPGCLCs that clusters together with non-hPGCLCs, have the authors taken this into consideration? Regarding the heatmaps, they do not seem very informative with respect to the clustering of differentially abundant metabolites or proteins between conditions. Maybe the authors should re-consider the clustering method or represent the data in a different manner."*

© 2026. Published by The Company of Biologists under the terms of the Creative Commons Attribution License (https://creativecommons.org/licenses/by/4.0/).

Answer: We have added additional information regarding the data treatment in the methodology (lines 463-475). We did not perceive a batch effect, considering that the replicates that are closer to each other are not consistent across cell groups. To elucidate this, the identification of the replicates (different symbols) has been added to the PCA plot, in figure 1. Furthermore, information regarding the collection of the iPSC samples has been added to the methodology (lines 430-432). For the heatmaps, we opted to order them based on p-value (we have also assessed ordering with VIP-score, but the result was similar). There is not a clear separation, particularly in the metabolite map. For that reason, we have included the volcano plots to better visualize the differences at protein and metabolite levels between groups.

Comment: *"From the GO analysis of the proteome (Figure 2) the main conclusion seems to be that hPGCLCs have a mayor cell adhesion/cytoskeleton remodelling compared to both iPSCs and non-hPGCLCs. This can also be observed when looking at the differentially expressed proteins in the volcano plot in Figure 3. However, the authors have not provided much discussion about this result. How does this correlate with previously published data, including transcriptomic data? Additionally, as a control for the proteome, the authors do not mention whether the main proteins that determine the hPGCLC fate become up-regulated in this cell type compared to iPCSCs/non-hPGCLCs. In fact, this type of analysis could be more informative than the immunostainings provided in Figure S1E or the qPCR in Figure S3."*

Answer: Regarding the functional enrichment analysis data, we opted to mainly center on the information tied to the energy metabolism since that is the main focus of this manuscript. Nonetheless, we have added some text on the discussion regarding the changes observed at the cytoskeleton remodelling/ cell adhesion level (lines 241-248). About the proteome data referring to the standard early PGC markers, just TFAP2C was detected in our cell groups (only in hPGCLCs). This is a common occurrence in untargeted proteomics studies, where detection can be affected by protein abundance, solubility and other variables. For this reason, the immunofluorescence experiment previous to the FACS sorting was used as validation of the success of the differentiation.

Comment: *"Regarding the protein changes, some metabolism-associated proteins come up as differentially expressed between h-PGCLCs and non-hPGCLCs. However, the fact that there are no significant differences in metabolites between h-PGCLCs and non-hPGCLCs seems to suggest both cell types are metabolically similar and therefore this feature is not a determinant of whether a differentiating cell acquires a PGC fate under this particular protocol. The authors argue that protein changes may precede metabolic changes, which could only be tested by assessing metabolism at later stages. However, an alternative explanation may be that the observed protein changes are not sufficient to impact metabolism. In this regard, it is noticeable that non of the proteome or metabolome results have been validated (by western blot/specific metabolic assays) in additional independent experiments."*

Answer: In this study we didn't detect differences at the metabolite levels between the differentiated hPGCLCs and non-hPGCLCs. We suggest that protein changes might precede metabolite changes, to the regulatory effect of such proteins (lines 241,242). The alternative explanation you mention might in fact be also part of this sequence of "events", since we could hypothesize that the observed changes in protein expression have not reached the threshold or level required to induce changes at metabolite level. To test this, a more mature state of in vitro derived hPGCLCs would need to be assessed, as mentioned in the discussion (lines 356,357). Since in this study we didn't have access to such material, we have added this as a limitation of this work (lines 354-358). Furthermore, regarding validation with additional experiments, we have now included data referent to a 2DG inhibitor essay that aligns with the HK1/HK2 findings of the proteomic data (lines 169-177, 283-293; Tables S13-16; Figure S4), see also answers to reviewer 1.

Comment: *"Finally, I find the discussion somewhat confusing as it does not clearly distinguish between hPGCLCs specific features and more general differentiation features (i.e. those that also affect non-hPGCLCs) and so it's not clear in what way their results could contribute to the improvement of hPGCLCs production."*

© 2026. Published by The Company of Biologists under the terms of the Creative Commons Attribution License (https://creativecommons.org/licenses/by/4.0/).

Answer: In the result section, we analysed to which cell group (iPSC, non-hPGCLC or both) we see a difference in metabolite or protein levels compared to hPGCLCs. In the discussion, we tied our argument to these results. To clarify which features are particular to the hPGCLCs, we have added to which cell groups (iPSC, non-hPGCLCs) we see the specific changes compared to hPGCLCs (lines 264,265,272,273,303,304,305). Furthermore, we have identified the hPGCLC-specific features in the end of the discussion (line 361).

Editor

Comment: *"I do not think that there is a requirement for additional experimentation. However, should the requested experimental results be available then please provide these in the revised manuscript."*

Answer: As validation of our -omics data, we have added a 2DG-inhibitor essay in combination with a human iPSC cell line harboring reporters for BLIMP1:tdTomato and TFAP2c:EGFP.

Because this glycolytic hexokinase inhibitor has been reported to have a higher effect in HK2, the isoform preferred by non-hPGCLCs (according to our proteomic data), the results support our proteomic data concerning HK1/HK2 expression. See reviewer 1 for elaborate explanation.

Comment: *"At this stage, we also ask you to ensure your manuscript complies with our formatting guidelines – please see our manuscript preparation guidelines for details."*

Answer: We have edited the manuscript to fit formatting guidelines of *Biology Open*. This included the inclusion of a running title (line 5) and keywords (lines 22,23), the reduction of the number of words in the abstract, the move of the additional methodology information from the supplements to the manuscript file (lines 435--474, 530-551) and the formatting of references to the journal style.

Comment: *"Please upload both a 'clean' version of your Word file, along with a highlighted version clearly showing where you have made changes in the revised manuscript. Please avoid using 'Track changes' in Word files as these are lost in PDF conversion."*

Answer: We have uploaded a version with the changes highlighted and a clean version, has requested.

<u>Second decision letter</u>

MS ID#: bio.062801R1

MS Title: Characterization of the metabolome and proteome of stem cell-derived human primordial germ cell-like cells: a multi-omics approach

Authors: Madalena Vaz Santos; Bauke Schomakers; Marta Llobet Ayala; Tara Jamali; Michel van Weeghel; Ans van Pelt; Callista Mulder; Geert Hamer

I am happy to tell you that your manuscript has been accepted for publication in Biology Open, pending our standard publication integrity checks. It was accepted on 26th August 2026.

© 2026. Published by The Company of Biologists under the terms of the Creative Commons Attribution License (https://creativecommons.org/licenses/by/4.0/).
